# Fourth Chromosome Resource Project: a comprehensive resource for genetic analysis in *Drosophila* that includes humanized stocks

**DOI:** 10.1093/genetics/iyad201

**Published:** 2023-11-20

**Authors:** Michael J Stinchfield, Brandon P Weasner, Bonnie M Weasner, David Zhitomersky, Justin P Kumar, Michael B O’Connor, Stuart J Newfeld

**Affiliations:** School of Life Sciences, Arizona State University, Tempe, AZ 85287-4501, USA; Department Biology, Indiana University, Bloomington, IN 47405, USA; Department Biology, Indiana University, Bloomington, IN 47405, USA; Department Genetics, Cell Biology and Development, University of Minnesota, Minneapolis, MN 55455, USA; Department Biology, Indiana University, Bloomington, IN 47405, USA; Department Genetics, Cell Biology and Development, University of Minnesota, Minneapolis, MN 55455, USA; School of Life Sciences, Arizona State University, Tempe, AZ 85287-4501, USA

**Keywords:** Activin/Inhibin, gene/protein traps, larval/adult brains

## Abstract

The fourth chromosome is the final frontier for genetic analysis in *Drosophila*. Small, heterochromatic, and devoid of recombination the fourth has long been ignored. Nevertheless, its long arm contains 79 protein-coding genes. The Fourth Chromosome Resource Project (FCRP) has a goal of facilitating the investigation of genes on this neglected chromosome. The project has 446 stocks publicly available at the Bloomington and Kyoto stock centers with phenotypic data curated by the FlyBase and FlyPush resources. Four of the five stock sets are nearly complete: (1) UAS.fly cDNAs, (2) UAS.human homolog cDNAs, (3) gene trap mutants and protein traps, and (4) stocks promoting meiotic and mitotic recombination on the fourth. Ongoing is mutagenesis of each fourth gene on a new FRT-bearing chromosome for marked single-cell clones. Beyond flies, FCRP facilitates the creation and analysis of humanized fly stocks. These provide opportunities to apply *Drosophila* genetics to the analysis of human gene interaction and function. In addition, the FCRP provides investigators with confidence through stock validation and an incentive via phenotyping to tackle genes on the fourth that have never been studied. Taken together, FCRP stocks will facilitate all manner of genetic and molecular studies. The resource is readily available to researchers to enhance our understanding of metazoan biology, including conserved molecular mechanisms underlying health and disease.

## Teaser

To fully understand the biology of an organism, one must know the function of all its genes. In *Drosophila melanogaster*, most genes on the 4th chromosome have been ignored. Among its 79 protein coding genes, only 42% have a mutant phenotype, while 94% have human homologs. Here, Stinchfield *et al*. report the Fourth Chromosome Resource Project, a collection of 445+ strains that provides the ability, previously unavailable, to study genes or mutations on the 4th.

## Introduction

**Fig. 1.**
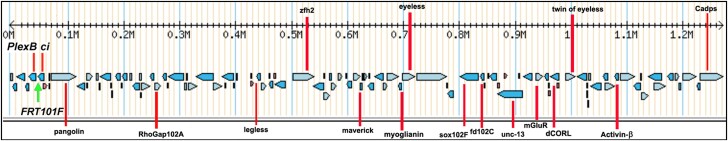
Genomic organization of the fourth long arm. The gridline reflects base pair numbering with the centromere to the left and telomere to the right. The arm contains 79 protein-coding genes (blue rectangles) and 26 very small noncoding RNAs (red rectangles). Several well-known genes such as *PlexinB*, *myo*, *eyeless*, *twin of eyeless*, and *Actβ* are identified with red lines. The insertion site of FRT101F for mitotic clones is indicated with a green arrow at 46,995 bp. See Goldsmith *et al*. (2022) for details regarding FRT101F.

To fully understand the biology of an organism, one must know the function of all its genes. In the premier genetic model organism *Drosophila melanogaster*, due to technical and biological barriers, most genes on its fourth chromosome were ignored (Fig. 1). Among these barriers are (1) extensive heterochromatic regions dispersed throughout the chromosome, (2) a transposon density roughly 5-fold higher than the other autosomes, and (3) the absence of recombination in both sexes. Prior to this project, only 42% of the 79 protein-coding genes on the long arm of the fourth had a mutation (Riddle and Elgin 2018). The remaining 58% were unstudied. The Fourth Chromosome Resource Project (FCRP) has significantly advanced the field by increasing the fraction with mutations to 91% and providing the first phenotypic data on the unstudied genes. From a wider perspective, what the FCRP provides to investigators is the genetic tools to study a gene or mutation on the fourth as if it were on any other chromosome.

The small fraction of fourth genes with mutations contrasts with a higher percentage of genes conserved in humans than the genome overall. There are 71 conserved genes (94%) that match to 713 unique human genes via Drosophila Integrative Ortholog Prediction Tool (DIOPT; Hu *et al* 2011) scores in FlyBase (Gramates *et al*. 2022). Among the human matches are components of several well-known signaling pathways including Hedgehog, Wingless, and TGF-β. There are also many neuronal genes on the fourth with human relatives such as *unc-13, Sox102F*, and *mGluR*. Overall, there are 10 gene families with multiple members encoded on the fourth chromosome (Table 1). These families include transcription factors, membrane proteins, kinases, and intercellular signals.

**Table 1.** Ten families with multiple members encoded on the fourth chromosome.

Families listed proximal to distal	Fourth proteins
ATP—ATP-binding cassette (ABC) transporters	JYalpha, Anne, ABCD1, PMCA
PLXN—Semaphorin transmembrane receptors	PlexA, PlexB
FOX—Forkhead box transcription factors	CG32006, Fd102C
ARF/ARL—ADP-ribosyltransferases	Arl4, Arf102
ZNF—Zinc-finger proteins	Dati, Pho, Zfh2
CAMK—Ca2++/calmodulin-dependent protein kinases	CAMKI, CAMKII
TGF-β—Transforming growth factor-β	Mav, Myo, Actβ
GRIK—Ionotropic glutamate receptors kainate type	Ekar, CG1115
PAX—Paired box transcription factors	Ey, Toy, Sv
LIM—Lin11, Isl1, and Mec3 domain transcription factors	CG33521, Zyx

Further, a majority of the matching human genes (68%) have a disease association. For example, *eyeless* belongs to the PAX/RAX family where the inherited loss of PAX6 leads to aniridia (Jordan *et al*. 1992) and somatic loss of RAX2 leads to age-related macular degeneration (Yang *et al*. 2015). Another example is *ankyrin* where mutations in ANK2 are the primary cause of congenital long QT syndrome, a potentially fatal heart condition (Hedley *et al*. 2009). Clearly, much interesting biology relevant to human health remains to be discovered on the fourth.

Recently, we reported stocks that enabled mitotic and meiotic recombination on the fourth (Goldsmith *et al*. 2022). Building on that advance, we expanded our efforts. Here, we report the many publicly available stocks generated by the FCRP, including those that can generate “humanized” stocks. These stocks have a genotype with a fly gene knocked out and a transgene with its human homolog expressed utilizing the fly gene's endogenous promoter. They facilitate the application of fly genetics to discover new knowledge of human gene interactions and functions. As exemplars, we report a pair of fly mutant rescue experiments that resulted in 2 distinct Inhibin-β humanized stocks. These stocks are valuable for testing new mechanistic hypotheses for INHBB and INHBC reported as a tumor suppressor or an oncogene in prostate cancer, respectively.

Overall, the goal of the FCRP is to provide stocks that allow anyone to study a gene on the fourth as easily as on any other chromosome. In addition to generating stocks, the FCRP provides investigators with confidence through validation and an incentive via phenotyping to tackle the many genes on the fourth that have never been studied. Taken together, FCRP stocks will facilitate all manner of genetic and molecular studies. The resource is readily available to researchers to enhance our understanding of metazoan biology, including conserved molecular mechanisms underlying health and disease.

## Materials and methods

### T2A.GAL4 and eGFP conversions then GFP validation in larval brains

#### Stocks

BL indicates a stock available from the Bloomington Stock Center (Whitworth 2019). To generate second and fourth chromosome double-balanced stocks for conversion crosses: *w; wg*^*Sp*^*/CyO* (BL8379), *yw; In(2LR)Gla wg*^*Gla*^*/SM6a* (BL6600), and *yw; TI{GMR-HMS04515}Gat*^*eya*^*/In(4)ci*^*D*^ (BL90852); for conversion, *P{y[ + mDint2] = Crey}1b, y M{vas-int.Dm}ZH-2A w* (BL60299), *yw; P{w[ + mC] = loxP(DH0)}7/CyO; TM2/TM6B e^s^ Tb* (BL80080), *yw; P{w[ + mC] = loxP(DH1)}7/CyO; TM2/TM6B e^s^ Tb* (BL80081), and *yw; P{w[ + mC] = loxP(DH2)}4/CyO; TM2/TM6B e^s^ Tb* (BL80082); and for validation of a conversion to a T2A.GAL4 gene trap, *w; P{w[ + mC] = UAS-GFP.nls}14* (BL4775).

#### MiMIC conversion

Crosses for MiMICs (Nagarkar-Jaiswel, DeLuca, *et al*. 2015; Nagarkar-Jaiswal, Lee, et al. 2015; Lee *et al*. 2018) and CRIMICs (Li-Kroeger *et al*. 2018, Kanca *et al*. 2019, 2022) are parallel to each other. For example, they require an insertion in a protein-coding region intron as both provide an artificial exon and both require a DoubleHeader donor construct matching the codon phase of the inserted gene's open reading frame (shown in the above stocks as DH0, DH1, or DH2). One difference is that MiMICs are marked with *y*+ while CRIMICs are marked with 3xP3-GFP. For MiMIC conversion crosses to T2A.GAL4 and eGFP, we followed Li-Kroeger *et al*. (2018) (see their Fig. 2, Supplementary Fig. 1; doi.org/10.7554/eLife.38709.007). We modified their scheme for fourth chromosome MiMICs in 2 ways: (1) by replacing the double-balanced chromosome 3 (TM2/TM6B) in each of the original 3 DoubleHeader stocks in cross 1 with a double-balanced fourth (*Gat*^*eya*^*/In(4)ci*^*D*^ derived from BL90852) and (2) in cross 3 males with the conversion genotype were crossed to females with UAS.nls-GFP homozygous on chromosome 2 carrying the double-balanced fourth. After scoring for *y*− indicating a conversion to either T2A.GAL4 or eGFP, cross 3 allowed us to immediately detect candidate T2A.GAL4 conversions in progeny. In our hands, efficiency was low with 5 vials of 2 or 3 males and 50 females each passed every day for 7 days producing roughly 1–5 male candidates. Single males were employed to create a balanced stock from each candidate, and then a single fly prep was followed by PCR to determine the orientation of the converted line. A complete list of converted stocks and their images is in Supplementary Table 1.

**Fig. 2.**
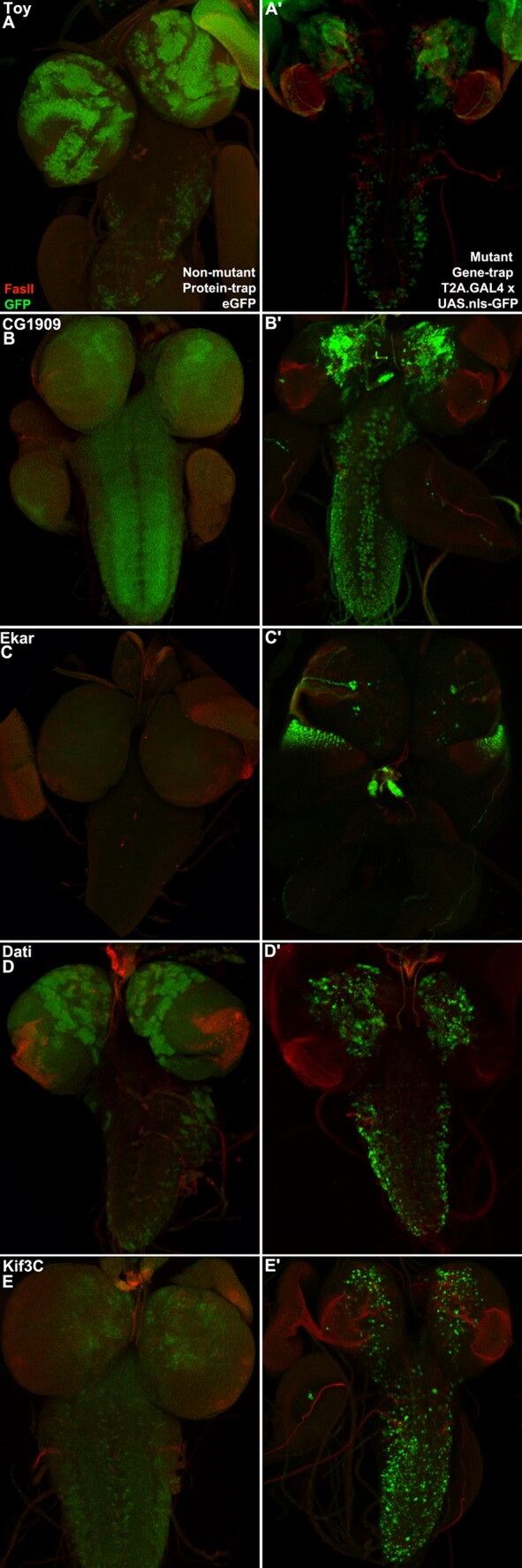
Identification of potential posttranscriptional regulation. Third instar larval brains with associated glands and imaginal disks reflecting GFP and FasII expression (*n* = 3 per genotype). Left column is antibody detection of eGFP in the endogenous protein. An eGFP allele is typically not a mutant. Right column is antibody detection of UAS.nls-GFP driven by a T2A.GAL4 cassette containing a stop codon truncating the inserted protein, a downstream IRES, and an initiator methionine for GAL4. A T2A.GAL4 allele is typically a mutant. a, b) *toy* and *CG1909* expression is roughly equivalent in their pair of brains. c) *Ekar* expression in the gene trap T2A.GAL4 brain and Ekar protein expression in the eGFP brain are clearly distinct. GFP is visible in the optic lobe, eye disks, and ring gland of the T2A.GAL4 brain but not in the eGFP brain. The distinction suggests the influence of posttranscriptional or posttranslational regulation. d, e) *dati* and *Kif3C* expression is roughly equivalent in their pair of brains.

#### PCR

We utilized 4 primers from Li-Kroeger *et al*. (2018) in 4 pairwise combinations to confirm DH conversion orientation (see their Supplementary File 1; page 1; last 4 lines for primer sequences; doi.org/10.7554/eLife.38709.017). Primers were as follows:

1. MiMIC 5′ forward: facing inward from the leading Minos element inverted repeat
2. MiMIC 3′ reverse: facing inward from the lagging Minos element inverted repeat
3. GFP forward: facing outward in the direction of GFP transcription
4. T2A.GAL4 reverse: facing outward in the opposite direction of T2A.GAL4 transcription

Logical consistency between the primer pairs producing products is informative. For example, if MiMIC 5′ forward and GFP forward produce a product as well as MiMIC 3′ reverse and T2A.GAL4 reverse, then GFP is adjacent to the 5′ end of the MiMIC. In this case, if the MiMIC is transcribed in the direction of the reading frame of the inserted gene, then GFP will be transcribed. If the MiMIC reading frame is transcribed in the opposite direction from the inserted gene's reading frame, then T2A.GAL4 will be transcribed. The PCR reactions were run in parallel, with a 50°C annealing temperature. Supplementary Fig. 1 shows results from *Activin-β* (*Actβ*) MI^14795^ conversion to T2A.GAL4 and eGFP. One line of each was kept for expression studies and deposited.

#### Immunohistochemistry

To validate T2A.GAL4 orientation in converted stocks and to gather the first gene expression data on the many fourth genes that have not been characterized, converted heterozygous males were crossed to females homozygous for UAS.nls-GFP and analyzed for GFP expression in third instar larval brains. Well-known FasII expression in a subset of neural membranes was employed as a counterstain in red to control for the detection of expression and for brain orientation. As described in Stinchfield *et al*. (2019), a mixture of male and female larvae was analyzed with chicken α-GFP (Abcam, ab13970), mouse α-FasII (DSHB 1D4), and matching Alexa Fluor secondary antibodies (Molecular Probes). Staining was followed by imaging in 2-µm sections with a Leica SP8 confocal microscope. To validate eGFP orientation in converted stocks and to gather the first protein expression data on fourth genes that have not been characterized, converted heterozygous females were analyzed for eGFP expression in third instar larval brains. Simple antibody detection was unsuccessful with eGFP, but we were successful with a minor modification to the Alexa Fluor 488 Tyramide SuperBoost Kit (Thermo Fisher, B40922) that includes a goat α-rabbit secondary. We employed rabbit α-GFP (Abcam, ab6556) and FasII. The modification was lengthening the TSA reaction to 45 min while rocking. Supplementary Fig. 2 shows images of GFP expression from the brains of *Actβ* T2A.GAL4 (BL93693) and Actβ eGFP (BL93676).

#### CRIMIC conversion

CRIMICs are T2A.GAL4 alleles by design and FCRP pioneered their conversion to eGFP with DoubleHeader crosses. In addition to carrying distinct markers, 2 other differences between MiMICS and CRIMICs are that CRIMICs are always inserted with their open reading frame matching that of the parent gene (rather than randomly for MiMICs), and there is no applicable PCR since the asymmetric Minos ends are absent. Employing the same DoubleHeader crossing scheme as for MiMICs with conversion in males in cross 3, candidate conversions in CRIMIC progeny were identified by loss of 3xP3-GFP in the eye. Similar efficiencies were obtained for MiMICs and CRIMIC conversions, and a stock was constructed from each single male candidate. Candidate stocks were tested for conversion to T2A.GAL4 by crossing to UAS.nls-GFP and the positives were discarded. The remaining stocks with no GFP in the eye were tested for eGFP. A complete list of converted stocks and their images is in Supplementary Table 1.

We soon realized that conversion of CRIMICs to DoubleHeader T2A.GAL4 eliminated the 3xP3-GFP marker and allowed GFP to again serve as a gene trap. Supplementary Fig. 3 shows images from larval brains expressing GFP: from 3xP3-GFP in the original *PlexA* CRIMIC T2A.GAL4, from a *PlexA* DoubleHeader T2A.GAL4 crossed to UAS.nls-GFP, and from a PlexA eGFP conversion. Only the latter two express GFP in the *PlexA* pattern. While T2A.GAL4-converted CRIMICs were not retained, if you have a need for a fourth CRIMIC converted to DoubleHeader T2A.GAL4, then contact the FCRP.

### HA-tagged UAS.fly cDNA stocks with validation in larval eye imaginal disks

#### Cloning

Multiple strategies for cloning cDNAs into pGW-HA.attB (Bischof *et al*. 2013, 2014) were employed depending upon the nature of the clones available from the Drosophila Genomics Resource Center (dgrc.bio.indiana.edu). One set of cDNAs was already cloned into pUAST-CFLAGHA-BD-PHI (https://fruitfly.org/EST/proteomics.shtml). This a UAS containing carboxy-terminal HA-tagged vector capable of chromosomal attP integration. These cDNAs were sequenced full-length open reading frames (Stapleton *et al*. 2002) that had their stop codons removed. The cDNA in each clone was sequenced for verification with recent *Drosophila* genome annotations (either 6.48 or 6.53). A second set of cDNAs was cloned into the Invitrogen Gateway pDONR plasmid. The complete cDNA sequence was obtained and verified, and then a Gateway LR reaction was utilized to shuttle the cDNA into pGW-HA.attB. A third set of cDNAs was cloned into non-Gateway plasmids. The complete cDNA sequence was obtained and verified and then amplified by PCR to generate a product with flanking Gateway recombination sequences. A Gateway BP reaction followed by an LR reaction sequentially moved the amplified cDNA PCR product into pDONR and then pGW-HA.attB. A fourth set of cDNAs had no reliable reagents. These cDNAs were synthesized by Synbio Technologies (Monmouth Junction, NJ, USA) and delivered as clones in the pUC57. From this clone, the cDNA sequence was amplified with Gateway primers and then recombined into pGW-HA.attB using the BP and LR reactions. pGW-HA.attB cDNA clones were injected at BestGene (Chino Hills, CA, USA). Each cDNA was inserted into a landing site on chromosome 2 (*yw; PBac{y[ + ]-attP-3B}VK00037*; BL9752) and chromosome 3 (*yw; PBac{y[ + ]-attP-3B}VK00033*; BL9750). For each cDNA at each landing site, a single homozygous line was deposited. For the subset of genes where it is known that a 3′ tag interferes with protein function (e.g. TGF-β family members), then no tag is present, and the genotype suffix is N instead of HA. A complete list of UAS.fly cDNA stocks is in Supplementary Table 2a.

#### Immunohistochemistry

Wandering third instar larval eye–antennal disk complexes were prepared as described (Spratford and Kumar 2014). They were then incubated in rat α-HA at 1:500 overnight at 4°C (Roche 3F10; Sigma-Aldrich, St. Louis, MO, USA). A subsequent incubation with FITC-conjugated donkey α-rat (712-005-153; Jackson ImmunoResearch, West Grove, PA, USA) and rhodamine phalloidin (R415; Thermo Fisher Scientific/Invitrogen, Waltham, MA, USA) was conducted at 1:100 for 2 h at 22°C. Eye disks were dissected and imaged on a Zeiss Axioplan.

### HA-tagged UAS.human cDNA stocks with validation in larval brains

#### Cloning

Human clones in Gateway donor vectors (pENTR 223.1 or pDONR 222, Invitrogen) were transferred into the vector pGW-HA.attB (same as fly cDNAs noted above) using Gateway LR clonase enzyme (11791019, Invitrogen). For clones not supplied in a Gateway-compatible system, the cDNA was PCR amplified from the parent plasmid using primers that hybridized to either end of the cDNA sequence with a Gateway sequence added to their 5′ ends. The amplified fragment was then cloned into pENTR 223.1 using Gateway BP clonase enzyme (11789020, Invitrogen). When a cDNA did not contain a stop codon at its 3′ end and one was biologically necessary, then an in-frame stop was engineered into the PCR 3′ reverse primer. After the clonase reaction, the mix was electroporated into DH5α *Escherichia coli* and plated with an antibiotic for the selection of transformants. The 5′ and 3′ ends of each human cDNA cloned into pGW-HA.attB were sequenced. Confirmed pGW-HA.attB cDNA clones were injected at BestGene. Each cDNA was inserted into a landing site on chromosome 2 (BL9752 or BL9723) and chromosome 3 (BL9750). For each cDNA at each landing site, a homozygous line was produced. One of the two stocks was deposited at Bloomington, and both were deposited in Kyoto. Note that when no tag is present, then the genotype suffix is N instead of HA. A complete list of UAS.human cDNA stocks is in Supplementary Table 2b.

#### Immunohistochemistry

third instar larvae carrying UAS.human cDNAs in pGW-HA.attB were crossed to *repo*.GAL4 (Sepp and Auld 2003). Larvae were inverted and fixed in PBST and 3.2% formaldehyde (Polysciences, Warrington, PA, USA) for 30 min at 22°C. Carcasses were washed 2 times with PBST and incubated overnight with 1:1,000 rat α-HA antibody (Roche 3F10) and mouse α-Repo (DSHB 8D12) at 4°C. Carcasses were washed 4 times for 30 min each, and then α-rat and α-mouse secondary antibodies conjugated to Alexa Fluor 488 and 633 (Molecular Probes) were added and incubated for 2 h at 22°C. Samples were washed in PBST; brains were dissected, transferred to 50% glycerol for clearing, mounted in 90% glycerol, and imaged with a Zeiss LSM.

### Rescue validates T2A.GAL4, UAS.fly, and UAS.human cDNAs leading to humanized stocks

#### Rescue stocks

*yw; Mi{DH.1}Act-β[MI14795-DH.GH-TG4.1]/In(4)ci* ^*D*^ (BL93693), *yw; P{w[ + mC] = UAS-Act-β.Z}3b2* (BL97108), *yw; PBac{y[ + mDint2] w[ + mC] = UAS-hINHBA.N}VK00002* (BL94337), *yw; PBac{y[ + mDint2] w[ + mC] = UAS-hINHBB.N}VK00002* (BL94338), *yw; PBac{y[ + mDint2] w[ + mC] = UAS-hINHBC.N}VK00002* (BL94339), *yw; PBac{y[ + mDint2] w[ + mC] = UAS-hINHBE.N}VK00002* (BL94340), and *w; P{w[ + mC] = tubP-GAL80^ts^}10; TM2/TM6B Tb* (BL7108).

#### Rescue experiments

First, a ubiquitous GAL80^ts^ transgene was recombined onto each of the 5 second chromosomes with a UAS.fly or UAS.human cDNA via standard methods. Recombinant chromosomes were kept balanced for consistency. Then, each of the recombinant second chromosomes was crossed into the homozygous lethal *Actβ* T2A.GAL4 background to create second and fourth chromosome double-balanced stocks. For each experiment, virgin heterozygous females were collected and crossed in groups of 20 with sibling virgin heterozygous males. Rescue crosses were set up at 5 temperatures from 18°C to 30°C. Progeny were scored daily for the fourth balancer with nonbalancer flies (homozygous for the T2A.GAL4 allele) considered rescued.

#### Immunohistochemistry

Mated adult females and males of varying ages from stocks with eGFP conversions for *Actβ* (*yw; Mi{DH.1}Actβ[MI14795-DH.PT-GFSTF.1]* BL93676) were collected and separated, and their brains were dissected in PBST and fixed in 4% formaldehyde in PBST. Brains were rinsed twice with PBST and then dehydrated stepwise into methanol with two 25% rinses, two 50% rinses, and then 100% for storage at −20°C. Before analysis, brains were rinsed in methanol, rehydrated with 50:50 methanol:PBST for 5 min, rinsed with PBST, and incubated in block solution (1% NGS in PBST). Primary antibodies were rabbit α-GFP and mouse α-Repo incubated overnight at 4°C. After a PBS rise, brains were incubated overnight at 4°C with secondary antibodies. After 4 PBS rinses, the TSA reaction was utilized. Brains were then rinsed in PBS, suspended in 90% glycerol/PBS, and stored overnight at 4°C before mounting and imaging.

### Mutagenized FRT101F chromosomes then FRT validation with MARCM clones

#### Stocks

*yw; TI{TI}FRT101F* (BL94596; abbreviated as FRT101F) and *yw; TI{FRT.Tub.GAL80.O}101F* (BL94598; abbreviated as FRT101F-Tub.GAL80), *yw P{ry[ + t7.2] = 70FLP}3* (BL6420), *yw; P{w[ + mC] = Act5C-GAL4}25FO1 P{w[ + mC] = UAS-GFP.U}2/CyO* (BL42726; abbreviated as *Actin5C*.GAL4-UAS.GFP with the original X in this stock replaced), and *yw; TI{TI}Crk[dsRed]/TI{GMR-HMS04515}Gat*^*eya*^ (BL90850). A complete list of fourth recombination stocks is found in Supplementary Table 3.

#### CRISPR

*zfh2* mutants were obtained using CRISPR mutagenesis. The guide RNA was designed with the O’Connor-Giles Target Finder program (https://flycrispr.org/target-finder/), and the sequence (5′-CGGCTGGCACGGGGGGAATC-3′) was selected as it has no predicted off-target sites. This guide was cloned into the expression vector pU6-BbsI-chiRNA (Gratz *et al*. 2013) and then injected by BestGene into a strain homozygous for *TI{TI}FRT101F* (Goldsmith *et al*. 2022). G0 progeny were crossed to *w; In(4)ci*^*D*^*/unc13-GFP.* Ten individual male and/or female F1s of the genotype w; FRT101F */In(4)ci^*D*^ from each G0 were backcrossed to *w; In(4)ci*^*D*^*/unc-13-GFP* flies. The F2 siblings from each cross with the genotype w; FRT101F */In(4)ci^*D*^ were self-crossed, and progeny was examined for the presence of only *In(4)ci*^*D*^ flies. Such vials contain a lethal mutation on the fourth chromosome. The current list of FRT101F mutant stocks is in Supplementary Table 3.

#### PCR

Genomic DNA was prepared, and PCR products were sequenced to identify insertions/deletions that could lead to frameshifts near the guide site. PCR and sequencing primers are as follows:

1. Forward 5′-AGGAAACATTGGAGATCCACATGAGG-3′
2. Reverse 5′-GCCCCAAGTAACTGCTGCA-3′

#### MARCM

For clones, we chose the allele *zfh2*^*51A*^. This is a 27-bp deletion that begins 1 bp beyond the splice donor for exon 6 (genomic coordinates 4:524907 to 4:524933). This results in the loss of 9 amino acids (597–605: QHPRLARGE) in zinc finger #3 (of 16) in all zfh2 protein isoforms. *zfh2*^*51A*^ is a hypomorphic allele with roughly 10% escapers. The method of Goldsmith and Newfeld (2023) was employed to generate MARCM clones. In brief, *yw; Actin5C.GAL4-UAS.GFP/CyO; FRT101F zfh^51A^/Gat*^*eya*^ males were mated to females homozygous for y*w hs-FLP; FRT101F-Tub.GAL80*; 25% of the progeny will contain the genotype capable of mitotic recombination *Actin.GAL4-UAS.GFP; FRT101F zfh^51A^*/*FRT101F-Tub.GAL80.* Eggs were collected in pairs of vials for 6 h at 25°C and then aged 4 h (egg age at heat shock was 4–10 h; mid-stage 9 to mid-stage 13 of development). A 1-h heat shock was conducted once at 37°C with 1 vial, and the eggs were returned to 25°C until larvae were collected. The other vial remained at 25°C as a negative control for clone formation.

#### Immunohistochemistry

Third instar larvae that have stopped wandering but prior to anterior spiracle eversion (122 h at 25°C for *yw*) were picked and brains dissected from GFP-positive larvae. Brains were fixed in 4% formaldehyde and rinsed and stored in methanol at −20°C overnight before staining. Primary antibodies were mouse α-Repo (DSHB 8D12) or rat α-Elav (DSHB 7E8A10) with chicken α-GFP (Abcam, ab13970). Secondaries were goat α-mouse, α-rat, or α-chicken Alexa Fluor 488 and 633 (Molecular Probes). Brains were mounted in 90% glycerol and imaged on a confocal employing 2-µm slices with the 20× objective or .5-µm slices with 100×.

## Results

### FCRP overview: 5 stock sets with their origins and utility

The FCRP is organized into 5 sets (Table 2). One set is genetic conversion of MiMIC and CRIMIC transgenes in protein-coding region introns into artificial exons creating either a T2A.GAL4 gene trap or an eGFP protein trap (164 stocks). Second is carboxy-terminal HA-tagged UAS.fly cDNAs in attP sites on chromosome 2 or 3 for gain-of-function studies (138 stocks). Third is carboxy-terminal HA-tagged UAS.human cDNAs in attP sites on chromosome 2 or 3 for gain-of-function studies with the top 2 human matches to conserved fourth chromosome genes (120 stocks). Fourth is FRT101F and related fourth recombination-facilitating chromosomes (12 stocks). Fifth is CRISPR mutations for each gene on the FRT101F chromosome for loss-of-function studies and MARCM clones (12 stocks). The goal is to provide “off the shelf” capability for genetic analysis on the fourth by community members at low cost.

**Table 2.** Summary of FCRP stocks available at Bloomington and Kyoto: 446.

T2A.GAL4 eGFP	UAS.fly cDNA	UAS.human cDNA	Recombination FRT101F	Mutations FRT101F
164	138	120	12	12

To advance the adoption of stocks from the resource, we provide the workflow for each of our chromosome sets and examples of their value to an investigator (Table 3). The creation of FRT101F and its related fourth recombination-facilitating chromosomes were previously reported (Goldsmith *et al*. 2022). We separate the genetic conversion of MiMICs and CRIMICs into separate T2A.GAL4 and GFP workflows to document their distinctive properties. We combine the UAS.fly and UAS.human cDNA sets into a single workflow as they utilize a common vector, attP sites, and experimental utility. Details for each set with examples follow.

**Table 3.** Workflow for each set of FCRP stocks.

Stock set citation	Starting point	Method or mechanism	Outcome	Utility	Stock combinations
T2A.GAL4, Li-Kroeger *et al*. (2018)	Intronic MiMIC or CRIMIC	Crosses create artificial exon with in-frame stop codon, IRES and GAL4 ORF	Gene trap: null mutation in target gene & GAL4 expressed in target pattern	1) Loss-of-function studies; 2) drive UAS.GFP for regulation studies	Compare to eGFP of same gene to identify posttranscriptional regulation
eGFP, Li-Kroeger *et al*. (2018)	Intronic MiMIC or CRIMIC	Crosses create artificial exon with in-frame eGFP ORF	Protein trap: eGFP-tagged target protein	Track protein expression: 1) Subcellular location; 2) ID target cells of secreted proteins	Compare different isoforms or reading frames of same gene to identify distinct roles
UAS.fly & UAS.human cDNA, Brand and Perrimon (1993)	cDNA clone or complete synthesis	Gateway cloning in 3′HA-tagged vector with attB sites into attP on II & III	HA-tagged cDNA for GAL4-driven expression	1) Gain-of-function studies; 2) epistasis pathway studies	Combined with T2A.GAL4 in rescue experiments led to humanized stocks
FRT101F mutant, Goldsmith *et al*. (2022)	FRT101F chromosome	Crosses with nosCas9 & ubi-gRNA then sequence progeny single candidate males	Loss-of-function mutant on a FRT101F chromosome	1) Identify phenotype of genomic mutant; 2) MARCM clone phenotypes	Add UAS cDNA to MARCM clones for rescue & epistasis studies

### T2A.GAL4 and eGFP conversions then GFP validation in larval brains

Justification for this set is that expression data are essential for understanding gene regulation and protein function. Initially, the FCRP applied a genetic conversion technique created by the Gene Disruption Project to a MiMIC transgene insertion in a protein-coding region intron (GDP; Bellen *et al*. 2011; Venken *et al*. 2011). In a single crossing scheme utilizing the DoubleHeader donor, FCRP created 2 types of converted alleles. One allele is a protein trap with an artificial exon encoding an in-frame eGFP tag (Nagarkar-Jaiswal, DeLuca, *et al*. 2015; Nagarkar-Jaiswal, Lee, et al. 2015). The endogenous protein is trapped visually by the incorporated eGFP. The other allele is a combination loss-of-function mutation with an in-frame gene trap artificial exon containing a T2A.GAL4 cassette (Lee *et al*. 2018). The T2A portion has a stop codon interrupting the translation of the endogenous protein followed by an internal ribosome entry site. The ribosome then reinitiates and translates GAL4. Expression of the endogenous transcript is trapped visually by GAL4-driven UAS.GFP.

The FCRP then extended the GDP DoubleHeader conversion method to CRIMIC transgenes. CRIMICs are inserted by the GDP via CRISPR into a protein-coding region intron for genes without a MiMIC (Li-Kroeger *et al*. 2018; Kanca *et al*. 2019, 2022). The T2A.GAL4 gene trap cassette built into a CRIMIC transgene is converted by the same DoubleHeader crossing scheme into an eGFP protein trap. For MiMICs, the screen for conversion is loss of an adult yellow cuticle marker. For CRIMICs, the screen for conversion is loss of *eyeless* promoter-driven GFP in the adult eye.

To date, 73 of the 79 protein-coding genes on the fourth (92%) have a T2A.GAL4 loss-of-function gene trap stock at Bloomington. Of these, stocks for 29 genes were generated by FCRP. Conversion of the remaining 6 intronless genes is being tackled with a new method by GDP. To date, 65 of the 79 protein-coding genes (83%) have an eGFP protein trap stock at Bloomington. Of these, stocks for 63 genes were generated by FCRP. The remaining 14 genes (7 intronless plus 7 resistant to DoubleHeader conversion) are being tackled with a new method by GDP. A summary of all fourth genes with T2A.GAL4 and eGFP stocks is in Supplementary Table 4.

The FCRP validates and phenotypes converted T2A.GAL4 and eGFP stocks in 2 ways. First is by noting if a converted allele is homozygous lethal to indicate whether that gene is essential. For 56% of the protein-coding genes on the fourth, their T2A.GAL4 insertion is homozygous lethal. Interestingly, this fraction of lethal genes is more than double the fraction of lethal genes in the overall genome at 23% (Ashburner *et al*. 1999). Alternatively, 22% of eGFP stocks are homozygous lethal indicating that the eGFP tag does not always interfere with function. For the 58% of unstudied genes on the fourth, FCRP data on homozygous lethality are their first phenotype.

The second validation is visualizing gene and protein trap expression with GFP. eGFP is detected in third instar larval brains with α-GFP. T2A.GAL4 is detected by crossing to UAS.nls-GFP and then displayed in the brains of progeny with α-GFP. The FCRP quickly recognized the value of comparing gene and protein trap expression phenotypes from the same gene, a “paired” expression analysis.

Comparing paired T2A.GAL4 and eGFP images can suggest hypotheses for gene regulation (Fig. 2). For example, *Ekar* is a candidate for regulation at the posttranscriptional (e.g. microRNA) or posttranslational (e.g. ubiquitin targeted degradation) levels based on the presence of GFP in the gene trap T2A.GAL4 larval brain but not in the protein trap eGFP brain. Images of GFP in larval brains for all FCRP T2A.GAL4 and eGFP stocks are available at FlyPush (the GDP website; https://flypush.research.bcm.edu/lab/index.html). Third instar larval brain GFP expression images were a focus of FCRP, for consistency with existing GDP larval brain images in FlyPush.

**Fig. 3.**
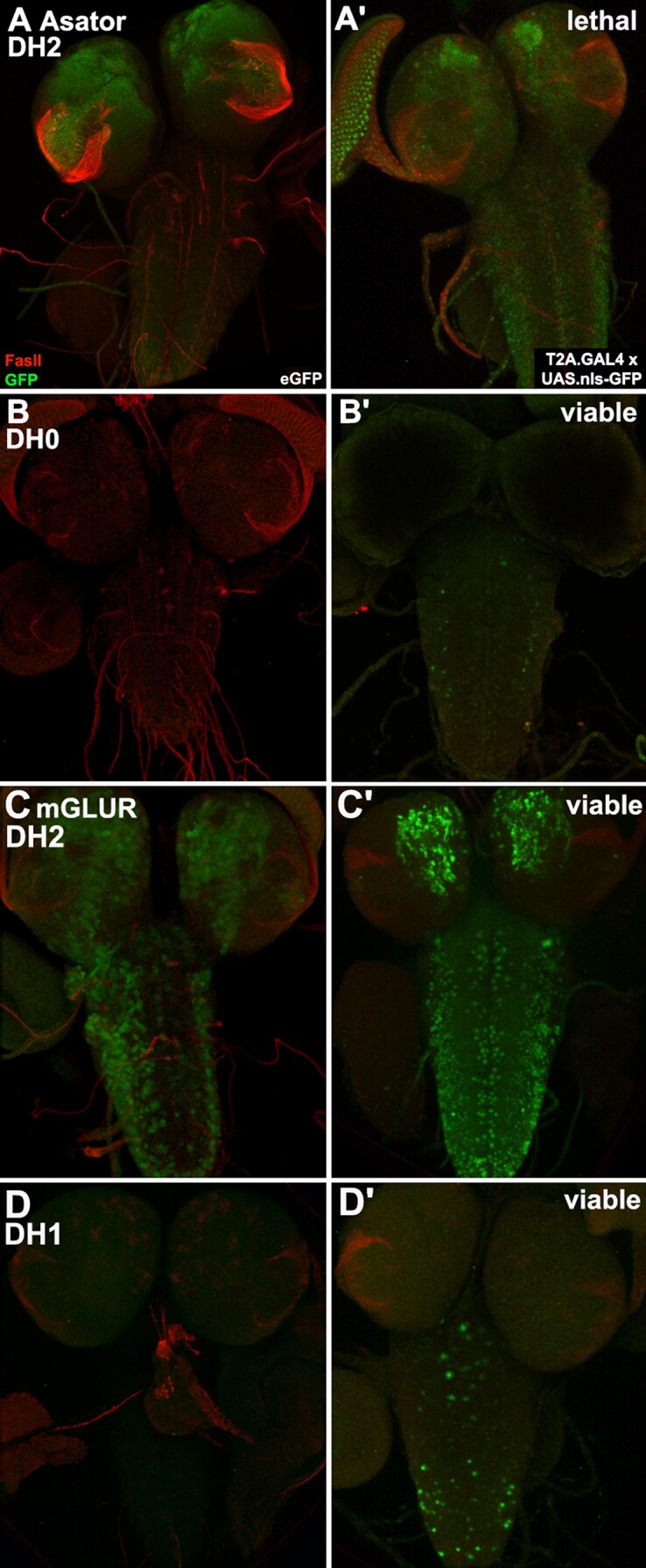
Identification of potential differential function for transcripts with different reading frames. Third instar brains with GFP and FasII expression (*n* = 2 per genotype). a, b) *Asator* eGFP and T2A.GAL4 conversions for frame DH2 (a, a′) and frame DH0 (b, b′) have distinct homozygous phenotypes (top right corner) and distinct expression pattens. c, d) *mGluR* eGFP and T2A.GAL4 conversions for frame DH2 (c, c′) and frame DH1 (d, d′) have similar homozygous phenotypes and distinct expression patterns.

Comparing paired T2A.GAL4 and eGFP images can also suggest hypotheses for alternative splicing (Fig. 3). There are 3 fourth genes that encode 2 proteins with partially distinct amino acid sequences due to splicing-induced changes in the transcript reading frame. For each, employing distinct DoubleHeader donors allowed the 2 reading frames to be converted independently to T2A.GAL4 and eGFP. For two of these genes, the T2A.GAL4 mutation phenotype in both frames is consistent, *mGluR* (homoviable) and *CG11155* (homolethal). Alternatively, *Asator* has 1 homoviable reading frame and 1 homolethal frame suggesting differential function. The T2A.GAL4 and eGFP expression patterns of the 2 *Asator* isoforms are different from each other but consistent with their homozygous phenotypes. *Asator* reading frame 2 (DH2) is homolethal and shows widespread larval brain GFP expression in both gene and protein traps. *Asator* reading frame 0 (DH0) is homoviable with only a few GFP-expressing cells in both gene and protein traps. FCRP-converted stocks facilitate further tests of specific hypotheses for the distinct functions of *Asator* alternative transcripts as well as for the other 2 genes.

**Fig. 4.**
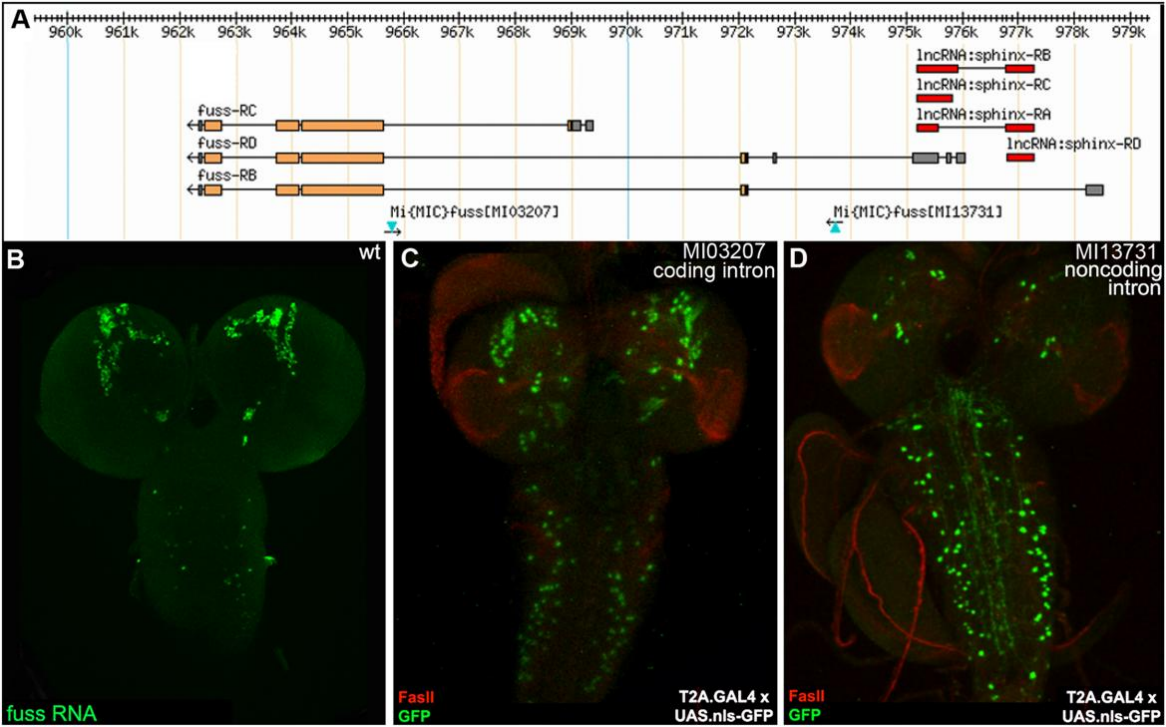
Identification of potential transcript-specific enhancers. a) The *fussel* (*fuss*) locus on the fourth with 3 transcripts and 2 MiMIC insertions plus the opposite strand lncRNA *sphinx* with 4 transcripts. MiMIC^13731^ is in a 5′-untranslated intron for *fuss* and near the transcription start of *sphinx*. MiMIC^03207^ is in a *fuss* coding intron. Conversion of both MiMICs and T2A.GAL4 was confirmed in the direction of *fuss* transcription by PCR. b–d) Third instar brains (*n* = 5 per genotype). b) *fuss* RNA in situ in wild type with a cDNA probe from the open reading frame. c) MiMIC^03207^-driven UAS.nls-GFP appears similar to *fuss* RNA. d) MiMIC^13731^-driven UAS.nls-GFP is distinct from *fuss* and perhaps reflects *sphinx* enhancers in the *fuss* 5′-untranslated intron.

Comparing paired T2A.GAL4 images can also suggest hypotheses for transcript-specific enhancers (Fig. 4). The *fussel* locus on the fourth (also known as dCORL; Takaesu *et al*. 2012; Tran *et al*. 2018) expresses 3 transcripts and contains the nested long noncoding RNA *sphinx* in a 5′-untranslated intron of its longest transcript. MiMIC^13731^ is inserted in this 5′-untranslated intron. MiMIC^03207^ is inserted in a coding intron downstream affecting all *fussel* transcripts. Conversion of both MiMICs to T2A.GAL4 was confirmed in the direction of *fussel* transcription by PCR. After crossing to UAS.nls-GFP, expression of GFP in both converted MiMIC larval brains was compared to *fussel* RNA in situ. The comparison revealed that the coding intron MiMIC looks similar to *fussel* RNA while the 5′-untranslated MiMIC does not. The difference suggests that the coding intron MiMIC reflects *fussel* enhancers and the 5′-untranslated MiMIC reflects *sphinx* enhancers in the *fussel* 5′-untranslated intron.

In summary, comparing paired T2A.GAL4 and eGFP images can suggest hypotheses for posttranscriptional gene regulation, for the differential function of alternative transcripts, and for the location of transcript-specific enhancers. We are certain there are many other scenarios where paired T2A.GAL4 and eGFP images will be valuable. FCRP T2A.GAL4 and eGFP stocks at Bloomington and Kyoto total 164 publicly available (Table 2). A complete list of FCR-converted stocks with stock center numbers plus the URL for their brain images is in Supplementary Table 1. Coverage of all fourth genes by T2A.GAL4 and eGFP stocks is in Supplementary Table 4.

### HA-tagged UAS.fly and UAS.human cDNA stocks with validation in larval disks and brains

Justification for the UAS.fly cDNA set is that gain-of-function (overexpression) studies complement loss-of-function (mutation) studies with the goal of understanding gene function. Overexpression studies also interrogate the integrity of mutant chromosomes via rescue experiments. Overexpression can be achieved using the GAL4-UAS system (Brand and Perrimon 1993). For these stocks, FCRP adopted a pGW-HA.attB vector from the UAS Orfeome project (Bischof *et al*. 2013, 2014) and the attP sites on chromosomes 2 and 3 employed by the GDP. Consistency with existing resources streamlined the adoption of FCRP stocks by the community.

**Fig. 5.**
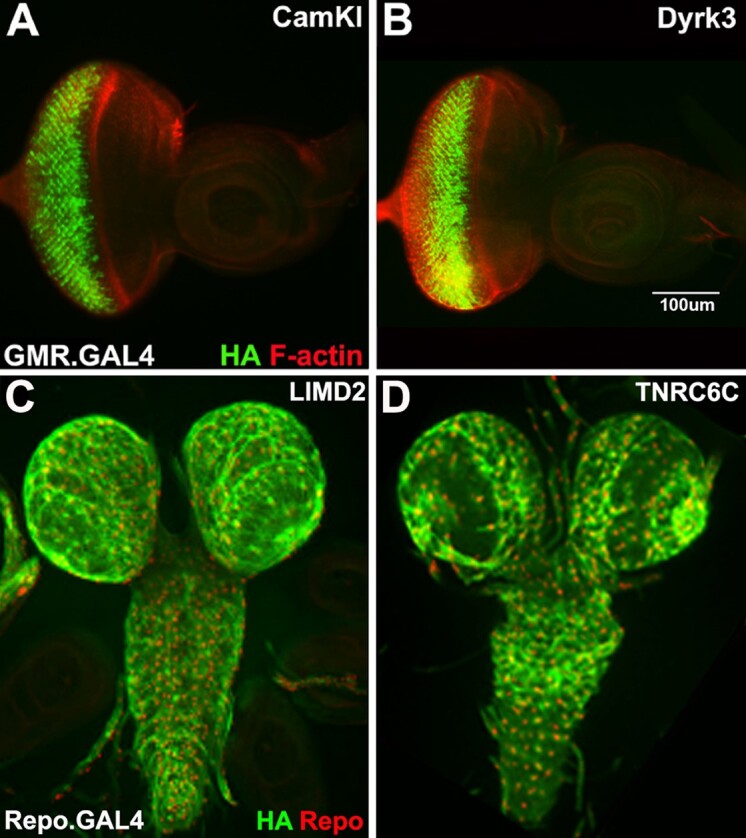
Expression validation for UAS.fly and UAS.human cDNA stocks. a, b) Third instar eye disks stained with α-HA (green) to detect the tagged cDNA and phalloidin (red) to mark F-Actin in mature ommatidia (*n* = 3 per genotype). GMR.GAL4 is driving either a) UAS.CAMKI or b) UAS.Dyrk3. HA expression is visible. c, d) Third instar larval brains stained with α-HA (green) and α-Repo (red) a nuclear glial marker (*n* = 2 per genotype). *repo*.GAL4 is driving either a) UAS.hLIMD2 related to *CG33521* on the fourth or b) UAS.hTNRC6C related to *gawky* on the fourth. HA expression is visible.

To date, 66 of the 79 protein-coding genes (84%) have at least 1 UAS.fly cDNA stock at Bloomington or published. FCRP has generated 56 UAS.fly cDNA stocks for 28 genes with insertions on chromosome 2 or 3. Plus 13 ORFeome UAS.fly cDNA stocks for fourth genes inserted on chromosome 3 were deposited in Bloomington and Kyoto at the request of the FCRP. UAS.fly cDNA stocks are validated in 2 ways. Expression is validated by driving each UAS.fly cDNA with GMR.GAL4 and imaging the HA tag in third instar eye disks (Fig. 5). Function is validated by rescuing homozygous T2A.GAL4 mutations in the parent gene (discussed below).

Justification for the UAS.human cDNA set is to allow *Drosophila* genetics to increase our understanding of human biology. FCRP investigators have a long history of studying human genes in flies. Examples of these studies revealed (1) the human gene's normal function (Marquez *et al*. 2001), (2) the disease-causing mechanism of mutant alleles from tumors (Takaesu *et al*. 2005), and (3) the precise human homolog when phylogenetic trees could not (Stinchfield *et al*. 2019). FCRP employs the same vector and insertion sites as UAS.fly cDNAs as well as the GDP method for identifying human cDNAs for cloning, scores generated by the DIOPT tool in FlyBase. A comprehensive analysis of DIOPT results showed that conserved fourth genes match 713 unique human genes. For efficiency, while ensuring maximum coverage, the FCRP is cloning the 2 best matching human cDNAs for each conserved fly gene into pGW-HA.attB. An exception is when there are more than 2 equivalent human matches to a single fourth gene (discussed below).

To date, 47 of the 74 conserved protein-coding genes on the fourth (64%) have 1 UAS.human cDNA stock at Bloomington, and 29 genes have 2 or more. FCRP has generated 60 UAS.human cDNA stocks for 31 genes with insertions on chromosome 2 or 3. UAS.human cDNA stocks are validated in 2 ways. Expression is validated by driving each UAS.human cDNA with *repo*.GAL4 and imaging the HA tag in third instar brains (Fig. 5). Function is validated by rescuing homozygous T2A.GAL4 mutations in the relevant fly gene (discussed below).

FCRP UAS.fly cDNA stocks at Bloomington and Kyoto total 138 stocks publicly available (Table 2). A complete list of FCRP UAS.fly cDNA stocks with stock center numbers is in Supplementary Table 2a. Coverage of all fourth genes with UAS.fly cDNA stocks is in Supplementary Table 4. FCRP UAS.human cDNA stocks at Bloomington and Kyoto total 120 publicly available (Table 2). A complete list of FCRP UAS.human cDNA stocks with stock center numbers is in Supplementary Table 2b. Coverage of all fourth genes with UAS.human cDNA stocks is in Supplementary Table 4.

### Rescue validates T2A.GAL4, UAS.fly, and UAS.human cDNAs leading to humanized stocks

Rescue experiments of homozygous lethal T2A.GAL4 mutations that validate stocks from the 3 sets above are important. A positive outcome (i.e. rescue of T2A.GAL4 lethality) shows that the T2A.GAL4 chromosome does not have a second-site mutation and that the UAS.fly or UAS.human cDNA is functional. For the subset of conserved genes on the fourth with 2 or more human counterparts, rescue studies have the additional benefit of identifying the true human homolog (e.g. Stinchfield *et al*. 2019).

In a pilot rescue experiment, FCRP employed the homozygous lethal T2A.GAL4 mutation in the TGF-β family member *Actβ*. Rescue crosses were planned with UAS.*Actβ* and its 4 closest human relatives INHBA, INHBB, INHBC, and INHBE (Wisotzkey and Newfeld 2020). Among the 4 human Inhibin-β proteins, the one with the most direct connection to Actβ is not discernable based on amino acid similarity. The relationships are also phylogenetically unresolved as shown in the tree in Supplementary Fig. 4. In this tree, all 4 Inhibin-β proteins are in a single cluster with *Actβ* at a bootstrap value of 0.94, with clusters above 0.85 considered statistically significant.

In preparation for the rescue crosses with these 5 genes, we noted that in paired images for *Actβ*, its T2A.GAL4 allele drove UAS.GFP at a higher intensity than endogenous Actβ protein expression reflected in eGFP (Supplementary Fig. 2). Thus, to compensate for the strength of T2A.GAL4 expression, a ubiquitous temperature-sensitive GAL80 (GAL80^ts^) transgene was added to the rescue genotype. GAL80 is a repressor of GAL4 function, and the logic was that GAL80^ts^ would provide greater control over UAS.*Actβ* expression than simply modulating GAL4 function directly via temperature. Rescue crosses were set between 18°C and 30°C. At 30°C, GAL80^ts^ is not functional and the parental stock was completely sterile due to overexpression of UAS.*Actβ*. At 18°C, GAL80^ts^ is fully functional, and in the parental stock, the *Actβ* mutant phenotype of pupal lethality was 100% penetrant. The hypothesis was that rescue of too much (sterility) and too little (lethality) Actβ would occur at an intermediate temperature.

**Fig. 6.**
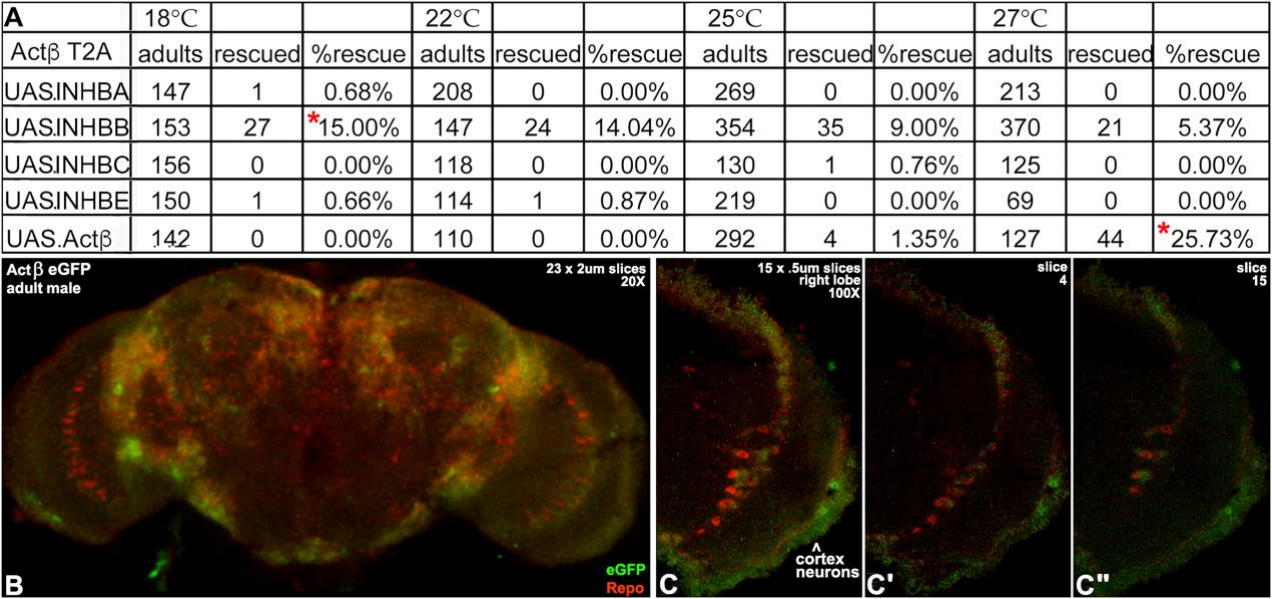
Rescue of *Actβ* T2A.GAL4 by human INHBB and detection of Actβ eGFP in the adult brain. a) Survival of *Actβ* T2A.GAL4 homozygous mutant adults with expression of UAS.*Actβ* or one of the four UAS human Inhibin-β transgenes. UAS.*Actβ* expressing *Actβ* T2A.GAL4 homozygous mutant adults survived best at 27°C (red star bottom right). UAS.INHBB expressing *Actβ* T2A.GAL4 homozygous mutant adults survived best at 18°C (red star upper left). b, c) Adult male brains (*n* = 4). b) Moderate size confocal stack at low magnification of a brain expressing Actβ eGFP (green) and Repo (red) a nuclear glial marker. Expression of Actβ eGFP is abundant near the periphery in cortex neurons. In the optic lobe, Actβ eGFP in neurons appears intertwined with the row of Repo expressing astrocyte-like glia in the chiasm. c) Small stack of the right lobe at higher magnification. Interdigitated Actβ eGFP and Repo are visible the optic lobe chiasm as is Actβ eGFP in cortex neurons. c′, c″) Confirmation of eGFP expression in both locations in a pair of slices from opposite ends of the stack.

Validation rescue of T2A.GAL4 homozygous lethality with UAS.*Actβ* was successful (Fig. 6a). This result shows that the *Actβ* cDNA is functional and that there are no second-site mutations on the *Actβ* T2A.GAL4 chromosome. The fraction of UAS.*Actβ*-rescued adults was 25.7%. This is nearly the fraction expected by Mendelian ratios (33%) and is equivalent to 78% rescued. A UAS.*Actβ* rescued *Actβ* mutant stock was created for future studies. Rescue of lethality with UAS.INHBB but none of the other human cDNAs was also successful. The fraction of UAS.INHBB rescued adults was 15.0% and is equivalent to 46% rescued. The rescue data also identified INHBB as the closest human relative to fly Actβ.

In a companion experiment, Actβ eGFP expression was examined in adult male brains (Fig. 6b and c). At low magnification, Actβ eGFP is widespread, including medial to the blood–brain barrier in cortex neurons. In the center of the optic lobe, Actβ eGFP is visible in neurons near a row of Repo expressing astrocyte-like glia in the chiasm. Higher magnification views of the optic lobe show that Repo and Actβ eGFP-expressing cells in the chiasm are intertwined. Repeating this eGFP analysis on adult brains of each sex at defined ages can provide age-dependent profiles of Actβ protein localization. These profiles can guide temperature shift experiments with rescued adults to examine the effect of loss of INHBB during aging on adult brain neurons and glia.

UAS.INHBB studies in rescued flies are also directly relevant to human disease. This is because exome sequencing has revealed a tumor-suppressive role for INHBB. Loss-of-function mutations were detected in endometrial and prostate cancer (Daly *et al*. 2022; Reader *et al*. 2022). However, the mechanism of action for INHBB in preventing prostate tumors is unknown. Based on the successful rescue of an *Actβ* mutation by UAS.INHBB, the INHBB rescued flies can be examined for cell cycle or cell signaling defects after age-dependent shifts to the nonpermissive temperature. These shifts phenocopy the loss of INHBB function similar to a somatic mutation. From this perspective, INHBB flies facilitate the testing of mechanistic hypotheses for INHBB in suppressing oncogenesis. Hypotheses can be tested cheaply and efficiently in humanized flies, before moving to more labor-intensive studies in mammals.

UAS.human cDNA rescue experiments can also expose unexpected redundancies in the human genome. A different set of crosses with the 4 human Inhibin-β proteins showed that UAS.INHBC will modestly rescue *myoglianin* (*myo*) T2A.GAL4 at 18°C (Table 4). The validation control UAS.*myo* rescued 100% of the expected homozygous T2A.GAL4 flies at the same temperature. INHBC is considered an oncogene in prostate cancer due to its effects when overexpressed (Ottley *et al*. 2017). UAS.INHBC-rescued flies provide an opportunity to test hypotheses for their oncogenic mechanism in temperature shift experiments.

**Table 4.** Myo T2A.GAL4 rescue by UAS*.myo* and UAS.INHBC is best at 18°C*.

	18°C			22°C			25°C			27°C		
*myo* T2A	adults	rescued	%rescue	adults	rescued	%rescue	adults	rescued	%rescue	adults	rescued	%rescue
UAS.INHBA	128	0	0.00%	130	1	0.01%	130	0	0.00%	133	0	0.00%
UAS.INHBB	n/a—parental lethal											
UAS.INHBC	218	12	*5.50%	236	9	3.81%	240	6	2.50%	211	10	4.74%
UAS.INHBE	106	1	0.01%	260	0	0.00%	183	0	0.00%	149	0	0.00%
UAS.*myo*	138	46	*33.33%	324	92	28.39%	577	192	33.27%	318	99	31.13%

Hypothesis testing can be conducted in rescued individuals derived from crosses. However, that approach is laborious (repeated need for the rescue cross) and inefficient (rescue genotypes are only 33% of offspring at best). Labor is saved and efficiency is achieved with the creation of humanized rescue stocks. These are perpetual stocks (no need for continuous crosses) that yield 100% rescued offspring. For example, the pilot experiment facilitated the creation of a UAS.INHBB rescued *Actβ* T2A.GAL4 mutant humanized stock. Analyses of humanized stocks that provide new information on the interactions and function of the human gene are limited only by the investigator's imagination. Genetic analyses could include epistasis or critical period studies via shift to the nonpermissive temperature. Biochemical analyses could include coimmunoprecipitation for protein–protein interactions or chromatin immunoprecipitation with massively parallel DNA sequencing (ChIP-Seq) for DNA binding. For example, a previous study employing flies expressing UAS.human Smad mutations identified in colon and pancreatic tumors revealed an unexpected gain-of-function mechanism for a specific subset of alleles (Takaesu *et al*. 2005).

### Mutagenized FRT101F chromosomes then FRT validation with MARCM clones

Justification for this set is to provide additional genomic loss-of-function data via the analysis of marked single-cell mutant clones for all fourth genes (MARCM; Lee and Luo 1999). Homozygosity for inherited genomic mutations affects all cells and provides information only on the first requirement for that gene. The creation of MARCM clones allows the investigation of genomic loss-of-function mutations in a GFP-expressing single-cell and its GFP-expressing descendants in a tissue and a time chosen by the investigator.

CRISPR was employed to place an FRT near the fourth centromere in polytene band 101F. The same method created the sibling FRT101F-Tub.GAL80 chromosome for MARCM clones. Combining these FRT chromosomes with heat-shock FLP, a neural-specific GAL4 and UAS.GFP created wild-type MARCM clones in specific cells in the brain (Goldsmith *et al*. 2022). Subsequently, the heat-shock protocol for MARCM was honed by employing a different set of FRT chromosomes and *Actin5C*.GAL4 to enable clones in any tissue at any time (Goldsmith and Newfeld 2023). FRT101F-related stocks at Bloomington and Kyoto total 12 publicly available (Table 2). A complete list of fourth recombination stocks with stock center numbers is in Supplementary Table 3.

Two other chromosomes that facilitate genetic analysis on the fourth, not created by FCRP but that we vetted and employed extensively, are new “balancer” fourth chromosomes (Nyberg *et al*. 2020). One has a dominant visible and homozygous lethal phenotype similar to Glazed that is easily scored in adults (BL90852). The other is a dominant visible and homozygous lethal ds-Red expressing chromosome that is scoreable in embryos and larvae (BL90851; both BL90850). These new balancers avoid complications of the 2 existing fourth balancers: reduced viability with *ey^D^* (Sturtevant 1936) and suppression/reversion of the vein phenotype with *ci*^*D*^ (Locke and Tartof 1994 ). These new balancers also avoid a CO_2_-sensitive lethal/sterile phenotype, primarily but not exclusively in males, uncovered in our lab when *ci*^*D*^ is paired with 6 T2A.GAL4 loss-of-function mutants (*ATPSynβ*, *crk*, *gw*, *myo*, *PlexA*, and *zfh2*).

To date, FCRP has mutagenized 56 genes with 21 still in the crossing scheme. Seventeen mutants are homozygous lethal, 20 are sequenced, and 7 were validated with MARCM clones. FRT101F-mutagenized stocks at Bloomington and Kyoto total 12 publicly available (Table 5). The current list of FRT101F-mutagenized stocks with stock center numbers is in Supplementary Table 3.

**Table 5.** FCRP CRISPR mutagenesis summary for 79 protein-coding genes.

Genes lacking gRNA	Genes undergoing mutagenesis	Genes awaiting sequencing	Genes with sequenced mutations	Lethal sequenced mutations	Viable sequenced mutations	Stocks deposited
4	21	48	20	8	12	12

**Fig. 7.**
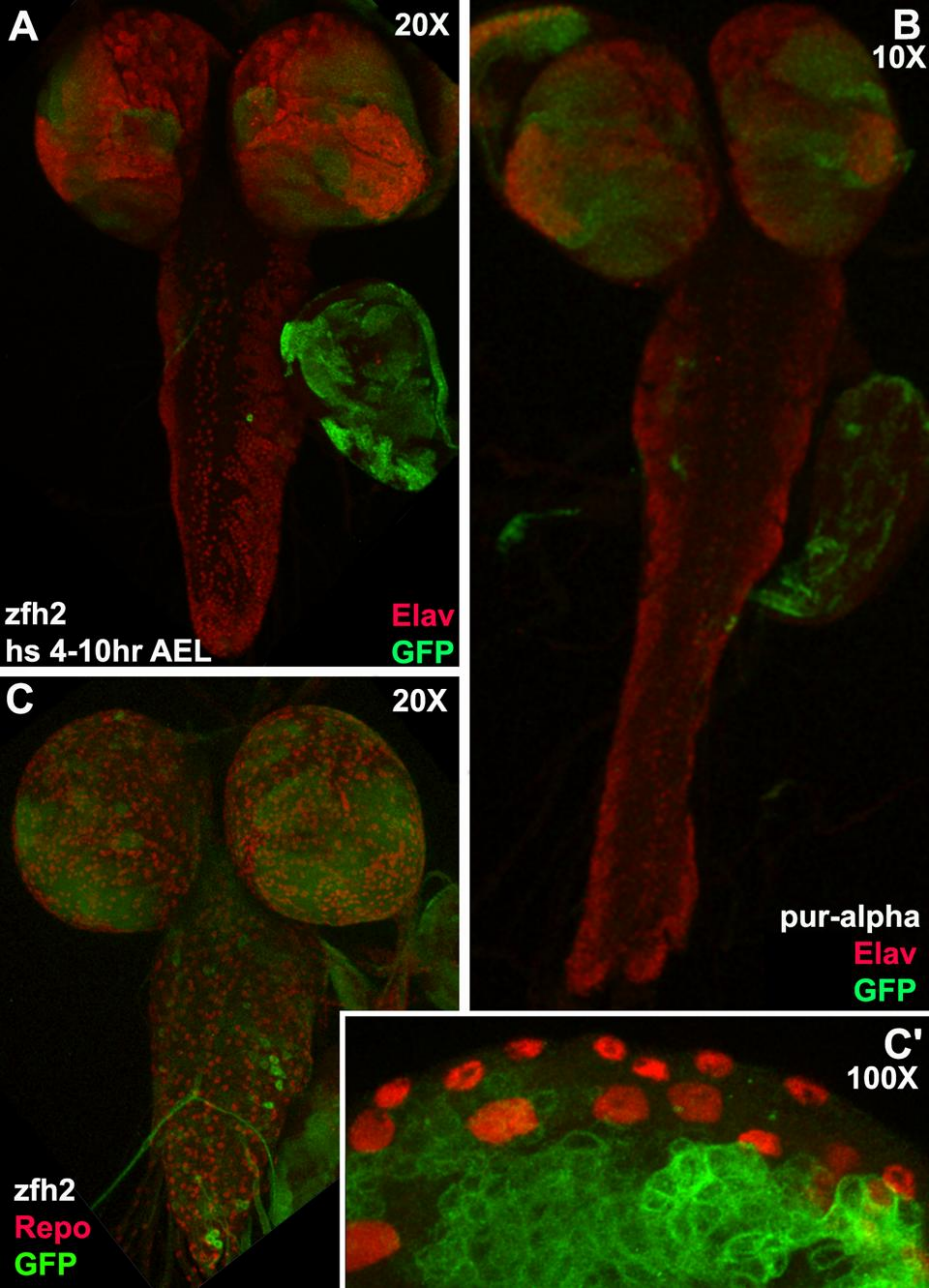
Validation of FRT function with MARCM clones employing mutagenized FRT101F chromosomes. Third instar larval brains (*n* = 6 per genotype). a) Numerous *zfh2*^*51A*^ clones marked with GFP (green) are visible in an Elav (red; nuclear neuronal marker) labeled brain and a wing disk. b) Numerous *Pur-α^B^* clones in the brain and a wing disk are visible. The ventral cord displays an extended phenotype potentially resulting from a failure to condense. Magnification was reduced to 10× from the usual 20× to capture the entire brain resulting in an image not to scale with panels a or c. c, c′) Numerous *zfh2*^*51A*^ clones are visible in Repo (red; nuclear glial marker) labeled brain lobes with a few clones in the ventral cord. High magnification view of a different *zfh2*^*51A*^ brain showing Repo labeled perineural glia on the surface and numerous clones in medially located cortex neurons.

Two FRT validation examples for mutants are shown in Fig. 7*. zfh2^51A^* is a 9-amino acid in-frame deletion within exon 6. The deletion removes a portion of zinc finger #3 (of 16) and functions as a hypomorphic allele. Mitotic recombination was induced in embryonic neuroblasts to produce multiple large MARCM clones in third instar larval brains. GFP-expressing clones demonstrate that FRT101F is functional after mutagenesis. *Pur-α^B^* is a frameshift-induced stop that eliminates the last 60% of the protein. MARCM clones of this allele induced in the embryo engender a mutant phenotype. The phenotype (*n* = 6) is a ventral cord that fails to condense, a phenotype recently reported for JNK signaling (Karkali *et al*. 2023).

## Discussion

While small, the importance of the fourth chromosome to the development and physiology of *D. melanogaster* is underscored by the fact that the fourth has a larger fraction of conserved genes and a larger fraction of essential genes than the genome as a whole. The FCRP has overcome the lack of recombination and the heterochromatic nature of the fourth with a goal of providing the tools to analyze genes on the fourth as if they were on any other chromosome. In an ongoing effort to achieve this goal, the FCRP generated and deposited 446 stocks at 2 stock centers.

To summarize FCRP progress, 91% of fourth genes now have a gene trap T2A.GAL4 mutation and 90% of genes have a protein trap eGFP tag. There are HA-tagged UAS.fly cDNA stocks for 84% of genes. There are HA-tagged UAS.human cDNA stocks for 64% of conserved fourth genes. Initial rescue experiments for fly T2A.GAL4 mutations with UAS.fly and UAS.human cDNAs have led to humanized stocks. These stocks enable studies of the human gene's normal interactions and functions as well as the opportunity to test the mechanism of action hypotheses for its disease associations. Mutations in each fourth gene are being generated on our FRT101F chromosome for loss-of-function studies with marked single-cell clones.

To ensure the *Drosophila* research community is aware of these new opportunities, FCRP takes multiple approaches. A list of our stocks is accessible on the Bloomington and Kyoto stock center websites by searching “Fourth Chromosome Resource Project.” All associated phenotypic data including homozygous lethality for mutants plus gene and protein expression patterns are accessible via the FlyBase and FlyPush resources for long-term curation and public availability. There are papers (Goldsmith *et al*. 2022) and presentations at the annual *Drosophila* research conference where input from the community is welcome. The FCRP also strives to bolster researchers with confidence in our stocks through validation. There is also an incentive to tackle a gene on the fourth that has never been studied based on our initial phenotyping. The notorious reputation of the fourth as a difficult place to work is being overcome.

The FCRP has begun to be recognized. In 2022, 311 FCRP stocks were ordered from Bloomington. That year, FCRP stocks for the 5 most popular fourth genes were ordered at a rate matching the orders for the top 3% of all Bloomington stocks. These are *toy* (12 orders), *PlexB* (13), *CaMKII* (14), *mGluR* (17), and *Actβ* (17). FCRP stock orders have a worldwide footprint with Bloomington reporting that in 2022, labs in 21 countries placed orders. In addition, the first dozen FCRP stock orders were recently reported by the Kyoto Stock Center.

In addition to bolstering confidence and providing incentive, the FCRP saves investigators money and time. Our focus on generating, validating, and phenotyping fourth chromosome stocks allows investigators to order them cheaply from a stock center. Our focus also allows for the generation of stocks at greater volume and at higher efficiency than in an individual lab. Overall, FCRP provides a range of stocks with varying capabilities for application to an investigator's favorite biological problem. Beyond the study of individual genes, resource projects like the FCRP have enabled countless genetic screens that led to fundamental discoveries.

Yet there is more work to do. The FCRP is moving forward in multiple areas. The first is to complete the unfinished sets of mutagenized FRT101F stocks and UAS.human cDNA stocks. The second is to create additional humanized stocks. For example, if UAS.MSTN rescued *myo* T2A.GAL4 mutants, then a subsequent humanized stock could be employed to test hypotheses for TGF-β-related muscle wasting (Tominaga and Suzuki 2019). The third was suggested to us, to complete the UAS.RNAi stock set begun by the Transgenic RNAi Project (Perkins *et al*. 2015). The fourth is to continue increasing confidence and providing incentive to analyze fourth chromosome genes by validating and phenotyping all fourth stocks whether they were made by the FCRP or not. For example, if a T2A.GAL4 mutant was created by GDP and the corresponding UAS.fly cDNA by the Orfeome project, the community would still benefit from reports of successful rescue.

Overall, the goal of the FCRP is to provide stocks that allow anyone to study a gene on the fourth as easily as on any other chromosome. In addition to generating stocks, the FCRP provides investigators with confidence through validation and an incentive via phenotyping to tackle the many genes on the fourth that have never been studied. Taken together, FCRP stocks will facilitate all manner of biochemical, genetic, and molecular studies. The resource is readily available to researchers to enhance our understanding of metazoan biology, including conserved molecular mechanisms underlying health and disease.

## Supplementary Material

iyad201_Supplementary_Data

## Data Availability

The authors affirm that all data necessary for confirming the conclusions of this article are present in this article, figures, tables, supplemental material, or publicly available databases such as FlyBase and FlyPush. Stocks are available from the Bloomington and Kyoto stock centers with lists of stock numbers for all FCRP stocks at each location in the supplemental material. Supplemental material available at GENETICS online.
