# Supplementary material for: Fourth Chromosome Resource Project: a comprehensive resource for genetic analysis in Drosophila that includes humanized stocks
Source: Genetics. 2023 Nov 20;226(2):iyad201. doi: 10.1093/genetics/iyad201 (PMC10847715; doi:10.1093/genetics/iyad201)
Supplement: iyad201_Supplementary_Data [file iyad201_supplementary_data.pdf]

Supplemental Information: 4 Figures and 4 Tables

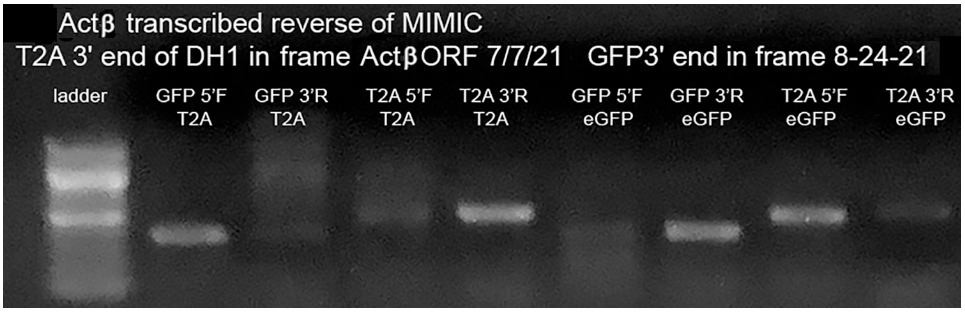

Fig. S1. Confirmation of *Actβ* MiMIC[14795] conversion to T2A.GAL4 and eGFP. PCR products in an agarose gel. Top line of text is the relative orientations of MiMIC and inserted gene transcription. Second line of text is our conclusion for the direction of conversion in the four lanes immediately below. Lanes numbered from left. Primer names above each lane with our conclusion below. Lane 1: 1kb ladder. Lanes 2-5: stock 2. Lane 2: GFP Forward + MiMIC 5' Forward = positive. Lane 3: GFP Forward + MiMIC 3' Reverse = negative. Lane 4: T2A.GAL4 Reverse + MiMIC 5’ Forward = negative. Lane 5: T2A.GAL4 Reverse + MiMIC 3’ Reverse = positive. Positive PCR in lanes 2 and 5 indicate conversion to T2A.GAL4. Lanes 6-9: stock 1. Lane 6: GFP forward + MiMIC 5’ Forward = negative. Line 7: GFP forward + MiMIC 3' Reverse = positive. Line 8: T2A.GAL4 reverse + MiMIC 5’ Forward = positive. Line 9: T2A.GAL4 reverse + MiMIC 3’ Reverse = negative. Positive PCR in lanes 7 and 8 indicate conversion to eGFP

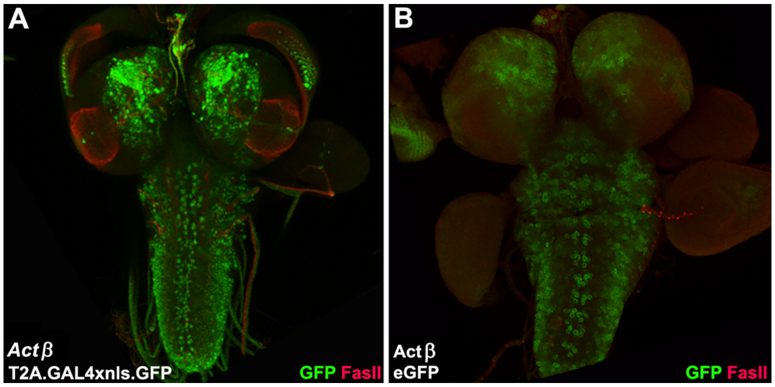

Fig. S2. ***Actβ*** **T2A.GAL4 and Actβ eGFP show similar spatial expression but greater intensity in T2A.GAL4.** 3rd instar brains expressing GFP from the converted MiMIC[14795] in *Actβ* and FasII. A) T2A.GAL4 driven UAS.nls-GFP is expressed in the nucleus of cells with *Actβ* transcription. B) eGFP detected via an artificial exon in the endogenous Actβ protein reflects the same spatial pattern except at lower intensity. Actβ eGFP appears cytoplasmic due to accumulation prior to secretion. Images take side by side with the same confocal settings.

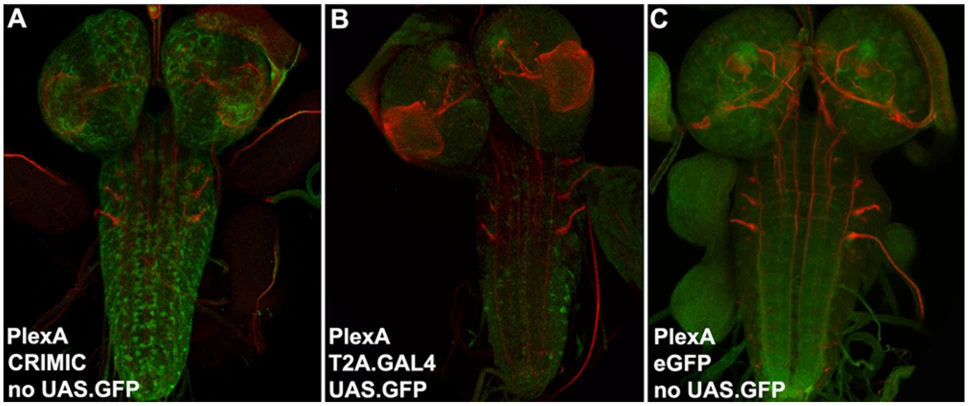

Fig S3. CRIMIC conversion to T2A.GAL4 eliminates 3xP3-GFP. 3rd instar brains expressing GFP and FasII. A) Parent *PlexA* CRIMIC (BL97187) expressing 3xP3-GFP alone. B) Conversion of *PlexA* CRIMIC to DH1 *PlexA* T2A.GAL4. Drives UAS.GFP as a gene trap (available upon request). C) Conversion of *PlexA* CRIMIC to DH1 *PlexA* eGFP. Acts as a protein trap (BL97750).

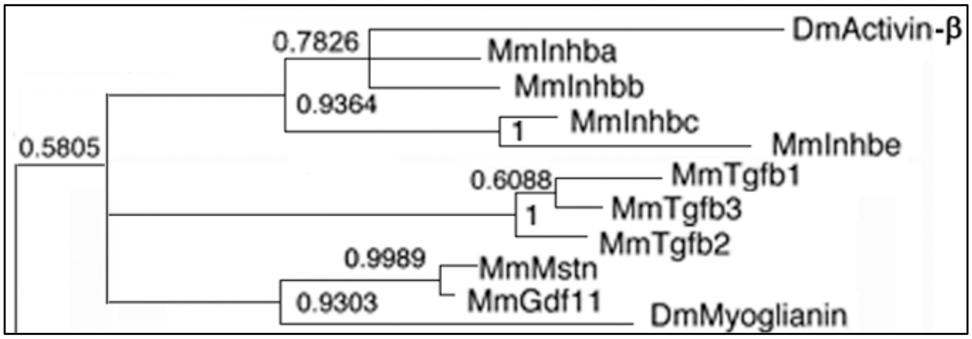

Fig. S4. Actβ is equally related to four and Myo to two mammalian proteins. Branch of a phylogenetic tree based on an alignment of mouse (Mm) TGF-β family proteins with fly (Dm) Actβ and Myo (from Fig. 9B of Wisotzkey & Newfeld 2020). Branch lengths to scale and numbers at nodes are bootstrap values indicating statistical confidence for that cluster. A bootstrap above 0.85 is significant. In this tree all four Inhibin-β proteins (INHBA, INHBB, INHBC, and INHBE) are in a single cluster with Actβ at a bootstrap of 0.9364. Two mammalian proteins (MSTN also known as GDF8 and GDF11) are in a single cluster with Myo at a bootstrap of 0.9303. Only rescue experiments can identify the Actβ and Myo mammalian homologs.

Table S1

FCRP: DoubleHeader converted T2A.GAL4 (TG4) and eGFP (GFSTF) stocks with center numbers and phenotypes			
Bloom #	Kyoto #	Genotype (82)	Flypush 3rd instar brain expression image url
93662	118860	y[1] w[1118];Mi{DH.1}RhoGAP102A[MI00706-DH.PT-GFSTF.1]	http://flypush.imgen.bcm.tmc.edu/pscreen/rmce/rmce.php?entry=RM10002
93663	118861	y[1] w[1118];Mi{DH.1}Ekar[MI02500-DH.PT-GFSTF.1]	http://flypush.imgen.bcm.tmc.edu/pscreen/rmce/rmce.php?entry=RM02111
93664	118862	y[1] w[1118];Mi{DH.0}CG1674[MI02589-DH.PT-GFSTF.0]	http://flypush.imgen.bcm.tmc.edu/pscreen/rmce/rmce.php?entry=RM10006
93665	118863	y[1] w[1118];Mi{DH.0}Kif3C[MI03059-DH.PT-GFSTF.0]	http://flypush.imgen.bcm.tmc.edu/pscreen/rmce/rmce.php?entry=RM10008
93666	118864	y[1] w[1118];Mi{DH.2}toy[MI03240-DH.PT-GFSTF.2]	http://flypush.imgen.bcm.tmc.edu/pscreen/rmce/rmce.php?entry=RM10010
93667	118865	y[1] w[1118];Mi{DH.1}CaMKII[MI03976-DH.PT-GFSTF.1]	http://flypush.imgen.bcm.tmc.edu/pscreen/rmce/rmce.php?entry=RM10013
93668	118866	y[1] w[1118];Mi{DH.2}CG1909[MI04521-DH.PT-GFSTF.2]	http://flypush.imgen.bcm.tmc.edu/pscreen/rmce/rmce.php?entry=RM10015
93669	118867	y[1] w[1118];Mi{DH.1}dati[MI04667-DH.PT-GFSTF.1]/In(4)ci[D], ci[D] pan[ciD]	http://flypush.imgen.bcm.tmc.edu/pscreen/rmce/rmce.php?entry=RM10017
93670	118868	y[1] w[1118];Mi{DH.1}Cadps[MI05877-DH.PT-GFSTF.1]	http://flypush.imgen.bcm.tmc.edu/pscreen/rmce/rmce.php?entry=RM10019
93671	118869	y[1] w[1118];Mi{DH.0}Nfl[MI06280-DH.PT-GFSTF.0]	http://flypush.imgen.bcm.tmc.edu/pscreen/rmce/rmce.php?entry=RM10021
93672	118870	y[1] w[1118];Mi{DH.1}MED26[MI06305-DH.PT-GFSTF.1]	http://flypush.imgen.bcm.tmc.edu/pscreen/rmce/rmce.php?entry=RM10023
93673	118871	y[1] w[1118];Mi{DH.0}Hcf[MI11162-DH.PT-GFSTF.0]	http://flypush.imgen.bcm.tmc.edu/pscreen/rmce/rmce.php?entry=RM10025
93674	118872	y[1] w[1118];Mi{DH.0}CG33521[MI13031-DH.PT-GFSTF.0]	http://flypush.imgen.bcm.tmc.edu/pscreen/rmce/rmce.php?entry=RM10027
93675	118873	y[1] w[1118];Mi{DH.0}Rnf11[MI14327-DH.PT-GFSTF.0]/In(4)ci[D], ci[D] pan[ciD]	http://flypush.imgen.bcm.tmc.edu/pscreen/rmce/rmce.php?entry=RM10029
93676	118874	y[1] w[1118];Mi{DH.1}Actbeta[MI14795-DH.PT-GFSTF.1]	http://flypush.imgen.bcm.tmc.edu/pscreen/rmce/rmce.php?entry=RM10031
93677	118875	y[1] w[1118];Mi{DH.1}PlexB[MI15559-DH.PT-GFSTF.1]	http://flypush.imgen.bcm.tmc.edu/pscreen/rmce/rmce.php?entry=RM10033
93678	118876	y[1] w[1118];Mi{DH.1}RhoGAP102A[MI00706-DH.GT-TG4.1]	http://flypush.imgen.bcm.tmc.edu/pscreen/rmce/rmce.php?entry=RM10001
93679	118877	y[1] w[1118];Mi{DH.1}Ekar[MI02500-DH.GT-TG4.1]	http://flypush.imgen.bcm.tmc.edu/pscreen/rmce/rmce.php?entry=RM10003
93680	118878	y[1] w[1118];Mi{DH.0}CG1674[MI02589-DH.GT-TG4.0]	http://flypush.imgen.bcm.tmc.edu/pscreen/rmce/rmce.php?entry=RM10004
93681	118879	y[1] w[1118];Mi{DH.0}Kif3C[MI03059-DH.GT-TG4.0]	http://flypush.imgen.bcm.tmc.edu/pscreen/rmce/rmce.php?entry=RM10007
93682	118880	y[1] w[1118];Mi{DH.2}toy[MI03240-DH.GT-TG4.2]/In(4)ci[D], ci[D] pan[ciD]	http://flypush.imgen.bcm.tmc.edu/pscreen/rmce/rmce.php?entry=RM10009
93683	118881	y[1] w[1118];Mi{DH.2}pan[MI03329-DH.GT-TG4.2]/TI{GMR-HMS04515}Gat[eya]	http://flypush.imgen.bcm.tmc.edu/pscreen/rmce/rmce.php?entry=RM10011
93684	118882	y[1] w[1118];Mi{DH.2}Asator[MI03458-DH.GT-TG4.2]	http://flypush.imgen.bcm.tmc.edu/pscreen/rmce/rmce.php?entry=RM10012
93685	118883	y[1] w[1118];Mi{DH.2}CG1909[MI04521-DH.GT-TG4.2]	http://flypush.imgen.bcm.tmc.edu/pscreen/rmce/rmce.php?entry=RM10014
93686	118884	y[1] w[1118];Mi{DH.1}dati[MI04667-DH.GT-TG4.1]/In(4)ci[D], ci[D] pan[ciD]	http://flypush.imgen.bcm.tmc.edu/pscreen/rmce/rmce.php?entry=RM10016
93687	118885	y[1] w[1118];Mi{DH.1}Cadps[MI05877-DH.GT-TG4.1]/In(4)ci[D], ci[D] pan[ciD]	http://flypush.imgen.bcm.tmc.edu/pscreen/rmce/rmce.php?entry=RM10018

93688	118886	y[1] w[1118];Mi{DH.0}Nfl[MI06280-DH.GT-TG4.0]/In(4)ci[D], ci[D] pan[ciD]	http://flypush.imgen.bcm.tmc.edu/pscreen/rmce/rmce.php?entry=RM10020
93689	118887	y[1] w[1118];Mi{DH.1}MED26[MI06305-DH.GT-TG4.1]/In(4)ci[D], ci[D] pan[ciD]	http://flypush.imgen.bcm.tmc.edu/pscreen/rmce/rmce.php?entry=RM10022
93690	118888	y[1] w[1118];Mi{DH.0}Hcf[MI11162-DH.GT-TG4.0]/In(4)ci[D], ci[D] pan[ciD]	http://flypush.imgen.bcm.tmc.edu/pscreen/rmce/rmce.php?entry=RM10024
93691	118889	y[1] w[1118];Mi{DH.0}CG33521[MI13031-DH.GT-TG4.0]	http://flypush.imgen.bcm.tmc.edu/pscreen/rmce/rmce.php?entry=RM10026
93692	118890	y[1] w[1118];Mi{DH.0}Rnf11[MI14327-DH.GT-TG4.0]/In(4)ci[D], ci[D] pan[ciD]	http://flypush.imgen.bcm.tmc.edu/pscreen/rmce/rmce.php?entry=RM10028
93693	118891	y[1] w[1118];Mi{DH.1}Actbeta[MI14795-DH.GT-TG4.1]/In(4)ci[D], ci[D] pan[ciD]	http://flypush.imgen.bcm.tmc.edu/pscreen/rmce/rmce.php?entry=RM10030
93694	118892	y[1] w[1118];Mi{DH.1}PlexB[MI15559-DH.GT-TG4.1]/In(4)ci[D], ci[D] pan[ciD]	http://flypush.imgen.bcm.tmc.edu/pscreen/rmce/rmce.php?entry=RM10032
94765	118893	y[1] w[1118]; TI{DH.1}pho[CR01824-DH.PT-GFSTF.1]	http://flypush.imgen.bcm.tmc.edu/pscreen/rmce/rmce.php?entry=RM20001
94766	118894	y[1] w[1118]; TI{DH.2}Tdg[CR01848-DH.PT-GFSTF.2]	http://flypush.imgen.bcm.tmc.edu/pscreen/rmce/rmce.php?entry=RM20004
94767	118895	y[1] w[1118]; Mi{DH.1}mGluR[MI02169-DH.PT-GFSTF.1]	http://flypush.imgen.bcm.tmc.edu/pscreen/rmce/rmce.php?entry=RM20008
94768	118896	y[1] w[1118]; Mi{DH.2}mGluR[MI02169-DH.PT-GFSTF.2]	http://flypush.imgen.bcm.tmc.edu/pscreen/rmce/rmce.php?entry=RM20006
94769	118897	y[1] w[1118]; Mi{DH.1}mGluR[MI02169-DH.GT-TG4.1]	http://flypush.imgen.bcm.tmc.edu/pscreen/rmce/rmce.php?entry=RM20007
94770	118898	y[1] w[1118]; Mi{DH.2}mGluR[MI02169-DH.GT-TG4.2]	http://flypush.imgen.bcm.tmc.edu/pscreen/rmce/rmce.php?entry=RM20005
94771	118899	y[1] w[1118]; Mi{DH.1}Pur-alpha[MI02955-DH.PT-GFSTF.1]	http://flypush.imgen.bcm.tmc.edu/pscreen/rmce/rmce.php?entry=RM20017
94772	118900	y[1] w[1118]; Mi{Trojan-GAL4.0}fuss[MI03207-TG4.0]	http://flypush.imgen.bcm.tmc.edu/pscreen/rmce/rmce.php?entry=RM20012
94773	118901	y[1] w[1118]; Mi{DH.2}pan[MI03329-DH.PT-GFSTF.2]	http://flypush.imgen.bcm.tmc.edu/pscreen/rmce/rmce.php?entry=RM20023
94774	118902	y[1] w[1118]; Mi{DH.0}Asator[MI03458-DH.PT-GFSTF.0]	http://flypush.imgen.bcm.tmc.edu/pscreen/rmce/rmce.php?entry=RM20021
94775	118903	y[1] w[1118]; Mi{DH.2}Asator[MI03458-DH.PT-GFSTF.2]	http://flypush.imgen.bcm.tmc.edu/pscreen/rmce/rmce.php?entry=RM20020
94776	118904	y[1] w[1118]; Mi{DH.0}Syt7[MI06277-DH.PT-GFSTF.0]	http://flypush.imgen.bcm.tmc.edu/pscreen/rmce/rmce.php?entry=RM20019
94777	118905	y[1] w[1118]; Mi{DH.1}bt[MI06578-DH.PT-GFSTF.1]	http://flypush.imgen.bcm.tmc.edu/pscreen/rmce/rmce.php?entry=RM20016
94778	118906	y[1] w[1118]; Mi{DH.2}Abcd1[MI11268-DH.PT-GFSTF.2]	http://flypush.imgen.bcm.tmc.edu/pscreen/rmce/rmce.php?entry=RM20013
94779	118907	y[1] w[1118]; Mi{Trojan-GAL4.un}fuss[MI13731-TG4.un-X]	http://flypush.imgen.bcm.tmc.edu/pscreen/rmce/rmce.php?entry=RM20011
94780	118908	y[1] w[1118]; TI{DH.0}Zyx[CR01526-DH.PT-GFSTF.0]	http://flypush.imgen.bcm.tmc.edu/pscreen/rmce/rmce.php?entry=RM20002
94781	118909	y[1] w[1118]; TI{DH.0}yellow-h[CR02253-DH.PT-GFSTF.0]	http://flypush.imgen.bcm.tmc.edu/pscreen/rmce/rmce.php?entry=RM20003
94782	118910	y[1] w[1118]; Mi{DH.0}Asator[MI03458-DH.GT-TG4.0]	http://flypush.imgen.bcm.tmc.edu/pscreen/rmce/rmce.php?entry=RM20022
94783	118911	y[1] w[1118]; Mi{DH.0}Eph[MI05205-DH.PT-GFSTF.0]	http://flypush.imgen.bcm.tmc.edu/pscreen/rmce/rmce.php?entry=RM20014
94784	118912	y[1] w[1118]; Mi{DH.1}dpr7[MI05719-DH.PT-GFSTF.1]	http://flypush.imgen.bcm.tmc.edu/pscreen/rmce/rmce.php?entry=RM20015
94785	118913	y[1] w[1118]; Mi{DH.2}PMCA[MI12515-DH.PT-GFSTF.2]	http://flypush.imgen.bcm.tmc.edu/pscreen/rmce/rmce.php?entry=RM20018
94786	118914	y[1] w[1118]; Mi{DH.2}ci[MI15343-DH.PT-GFSTF.2]	http://flypush.imgen.bcm.tmc.edu/pscreen/rmce/rmce.php?entry=RM20009

94787	118915	y[1] w[1118]; Mi{DH.2}ci[MI15343-DH.GT-TG4.2]/TI{GMR-HMS04515}Gat[eya]	http://flypush.imgen.bcm.tmc.edu/pscreen/rmce/rmce.php?entry=RM20010
97733	118953	y[1] w[1118]; TI{DH.2}sv[CR00370-DH.PT-GFSTF.2]/In(4)ci[D], ci[D] pan[ciD]	https://flypush.research.bcm.edu/pscreen/rmce/rmce.php?entry=RM20024
97734	118954	y[1] w[1118]; TI{DH.1}CG31999[CR01673-DH.PT-GFSTF.1]	https://flypush.research.bcm.edu/pscreen/rmce/rmce.php?entry=RM20025
97735	118955	y[1] w[1118]; TI{DH.1}Rad23[CR01823-DH.PT-GFSTF.1]	https://flypush.research.bcm.edu/pscreen/rmce/rmce.php?entry=RM20026
97736	118956	y[1] w[1118]; TI{DH.2}CG31998[CR01849-DH.PT-GFSTF.2]	https://flypush.research.bcm.edu/pscreen/rmce/rmce.php?entry=RM20027
97737	118957	y[1] w[1118]; TI{DH.2}CG32017[CR01850-DH.PT-GFSTF.2]	https://flypush.research.bcm.edu/pscreen/rmce/rmce.php?entry=RM20028
97738	118958	y[1] w[1118]; TI{DH.0}Ank[CR01879-DH.PT-GFSTF.0]	https://flypush.research.bcm.edu/pscreen/rmce/rmce.php?entry=RM20029
97739	118959	y[1] w[1118]; TI{DH.1}ey[CR02257-DH.PT-GFSTF.1]	https://flypush.research.bcm.edu/pscreen/rmce/rmce.php?entry=RM20030
97740	118961	y[1] w[1118]; TI{DH.0}Arl4[CR02574-DH.PT-GFSTF.0]	https://flypush.research.bcm.edu/pscreen/rmce/rmce.php?entry=RM20031
97741	118962	y[1] w[1118]; TI{DH.1}CaMKI[CR02670-DH.PT-GFSTF.1]	https://flypush.research.bcm.edu/pscreen/rmce/rmce.php?entry=RM20032
97742	118963	y[1] w[1118]; TI{DH.0}Slip1[CR02779-DH.PT-GFSTF.0]	https://flypush.research.bcm.edu/pscreen/rmce/rmce.php?entry=RM20033
97743	118964	y[1] w[1118]; TI{DH.1}anne[CR70036-DH.PT-GFSTF.1]	https://flypush.research.bcm.edu/pscreen/rmce/rmce.php?entry=RM20034
97744	118965	y[1] w[1118]; TI{DH.1}apolpp[CR70471-DH.PT-GFSTF.1]/In(4)ci[D], ci[D] pan[ciD]	https://flypush.research.bcm.edu/pscreen/rmce/rmce.php?entry=RM20035
97745	118966	y[1] w[1118]; TI{DH.1}CG31997[CR70475-DH.PT-GFSTF.1]	https://flypush.research.bcm.edu/pscreen/rmce/rmce.php?entry=RM20036
97746	118967	y[1] w[1118]; TI{DH.0}CG33941[CR70477-DH.PT-GFSTF.0]	https://flypush.research.bcm.edu/pscreen/rmce/rmce.php?entry=RM20037
97747	118968	y[1] w[1118]; TI{DH.0}gw[CR70482-DH.PT-GFSTF.0]/TI{GMR-HMS04515}Gat[eya]	https://flypush.research.bcm.edu/pscreen/rmce/rmce.php?entry=RM20038
97748	118969	y[1] w[1118]; TI{DH.0}JYalpha[CR70483-DH.PT-GFSTF.0]/In(4)ci[D], ci[D] pan[ciD]	https://flypush.research.bcm.edu/pscreen/rmce/rmce.php?entry=RM20039
97749	118970	y[1] w[1118]; TI{DH.0}PIP4K[CR70484-DH.PT-GFSTF.0]/In(4)ci[D], ci[D] pan[ciD]	https://flypush.research.bcm.edu/pscreen/rmce/rmce.php?entry=RM20040
97750	118971	y[1] w[1118]; TI{DH.1}PlexA[CR70485-DH.PT-GFSTF.1]/TI{GMR-HMS04515}Gat[eya]	https://flypush.research.bcm.edu/pscreen/rmce/rmce.php?entry=RM20041
97751	118972	y[1] w[1118]; TI{DH.2}zfh2[CR70564-DH.PT-GFSTF.2]/TI{GMR-HMS04515}Gat[eya]	https://flypush.research.bcm.edu/pscreen/rmce/rmce.php?entry=RM20042
97752	118973	y[1] w[1118]; TI{DH.1}Gyf[CR70565-DH.PT-GFSTF.1]/In(4)ci[D], ci[D] pan[ciD]	https://flypush.research.bcm.edu/pscreen/rmce/rmce.php?entry=RM20043
97753	118974	y[1] w[1118]; TI{DH.1}4E-T[CR92248-DH.PT-GFSTF.1]	https://flypush.research.bcm.edu/pscreen/rmce/rmce.php?entry=RM20044
97754	118975	y[1] w[1118]; TI{DH.1}lgs[CR92249-DH.PT-GFSTF.1]	https://flypush.research.bcm.edu/pscreen/rmce/rmce.php?entry=RM20045
97755	118976	y[1] w[1118]; Mi{DH.0}fuss[MI03207-DH.PT-GFSTF.0]	https://flypush.research.bcm.edu/pscreen/rmce/rmce.php?entry=RM20046
97756	118977	y[1] w[1118]; Mi{DH.1}CaMKII[MI03976-DH.GT-TG4.1]/TI{GMR-HMS04515}Gat[eya]	https://flypush.research.bcm.edu/pscreen/rmce/rmce.php?entry=RM20047
600215	118960	y[1] w[1118]; TI{DH.1}myo[CR02262-DH.PT-GFSTF.1]/TI{GMR-HMS04515}Gat[eya]	https://flypush.research.bcm.edu/pscreen/rmce/rmce.php?entry=RM20048

Table S2A

FCRP: UAS.fly cDNA stocks with Bloomington and Kyoto center numbers		
**Bloom #**	**Kyoto #**	**Genotype (69)**
93837	118756	y[1] w[1118]; PBac{y[+mDint2] w[+mC]=UAS-CaMKI.HA}VK00037
93838	118757	y[1] w[1118]; PBac{y[+mDint2] w[+mC]=UAS-CaMKI.HA}VK00033
93839	118758	y[1] w[1118]; PBac{y[+mDint2] w[+mC]=UAS-ND-49.HA}VK00037
93840	118759	y[1] w[1118]; PBac{y[+mDint2] w[+mC]=UAS-ND-49.HA}VK00033
93841	118760	y[1] w[1118]; PBac{y[+mDint2] w[+mC]=UAS-Arl4.HA}VK00037
93842	118761	y[1] w[1118]; PBac{y[+mDint2] w[+mC]=UAS-Arl4.HA}VK00033
93843	118762	y[1] w[1118]; PBac{y[+mDint2] w[+mC]=UAS-CG1674.HA}VK00037
93844	118763	y[1] w[1118]; PBac{y[+mDint2] w[+mC]=UAS-CG1674.HA}VK00033
93845	118764	y[1] w[1118]; PBac{y[+mDint2] w[+mC]=UAS-Kif3C.HA}VK00037
93846	118765	y[1] w[1118]; PBac{y[+mDint2] w[+mC]=UAS-Kif3C.HA}VK00033
93847	118766	y[1] w[1118]; PBac{y[+mDint2] w[+mC]=UAS-Dyrk3.HA}VK00037
93848	118767	y[1] w[1118]; PBac{y[+mDint2] w[+mC]=UAS-Dyrk3.HA}VK00033
93849	118768	y[1] w[1118]; PBac{y[+mDint2] w[+mC]=UAS-Cals.HA}VK00037
93850	118769	y[1] w[1118]; PBac{y[+mDint2] w[+mC]=UAS-Cals.HA}VK00033
93851	118770	y[1] w[1118]; PBac{y[+mDint2] w[+mC]=UAS-Rnf11.HA}VK00037
93852	118771	y[1] w[1118]; PBac{y[+mDint2] w[+mC]=UAS-Rnf11.HA}VK00033
93853	118772	y[1] w[1118]; PBac{y[+mDint2] w[+mC]=UAS-CG31997.HA}VK00037
93854	118773	y[1] w[1118]; PBac{y[+mDint2] w[+mC]=UAS-CG31997.HA}VK00033
93855	118774	y[1] w[1118]; PBac{y[+mDint2] w[+mC]=UAS-Arf102F.HA}VK00037
93856	118775	y[1] w[1118]; PBac{y[+mDint2] w[+mC]=UAS-Arf102F.HA}VK00033
94867	118776	y[1] w[1118]; PBac{y[+mDint2] w[+mC]=UAS-Abcd1.HA}VK00037
94868	118777	y[1] w[1118]; PBac{y[+mDint2] w[+mC]=UAS-Abcd1.HA}VK00033
94869	118778	y[1] w[1118]; PBac{y[+mDint2] w[+mC]=UAS-4E-T.HA}VK00037
94870	118779	y[1] w[1118]; PBac{y[+mDint2] w[+mC]=UAS-4E-T.HA}VK00033
94871	118780	y[1] w[1118]; PBac{y[+mDint2] w[+mC]=UAS-CG31999.HA}VK00037
94872	118781	y[1] w[1118]; PBac{y[+mDint2] w[+mC]=UAS-CG31999.HA}VK00033
94873	118782	y[1] w[1118]; PBac{y[+mDint2] w[+mC]=UAS-CG11155.HA}VK00037
94874	118783	y[1] w[1118]; PBac{y[+mDint2] w[+mC]=UAS-CG11155.HA}VK00033
94875	118784	y[1] w[1118]; PBac{y[+mDint2] w[+mC]=UAS-Ekar.HA}VK00037
94876	118785	y[1] w[1118]; PBac{y[+mDint2] w[+mC]=UAS-Ekar.HA}VK00033
94877	118786	y[1] w[1118]; PBac{y[+mDint2] w[+mC]=UAS-CG32017.HA}VK00037
94878	118787	y[1] w[1118]; PBac{y[+mDint2] w[+mC]=UAS-CG32017.HA}VK00033
94879	118788	y[1] w[1118]; PBac{y[+mDint2] w[+mC]=UAS-lgs.HA}VK00037
94880	118789	y[1] w[1118]; PBac{y[+mDint2] w[+mC]=UAS-lgs.HA}VK00033
99865		y[1] w[1118]; PBac{y[+mDint2] w[+mC]=UAS-yellow-h.HA}VK00037
99866		y[1] w[1118]; PBac{y[+mDint2] w[+mC]=UAS-yellow-h.HA}VK00033
99867		y[1] w[1118]; PBac{y[+mDint2] w[+mC]=UAS-Gat.HA}VK00037
99868		y[1] w[1118]; PBac{y[+mDint2] w[+mC]=UAS-Gat.HA}VK00033
99869		y[1] w[1118]; PBac{y[+mDint2] w[+mC]=UAS-CG1909.HA}VK00037
99870		y[1] w[1118]; PBac{y[+mDint2] w[+mC]=UAS-CG1909.HA}VK00033
99871		y[1] w[1118]; PBac{y[+mDint2] w[+mC]=UAS-CG11076.HA}VK00037

99872		y[1] w[1118]; PBac{y[+mDint2] w[+mC]=UAS-CG11076.HA}VK00033
99873		y[1] w[1118]; PBac{y[+mDint2] w[+mC]=UAS-Mpv17.HA}VK00037
99874		y[1] w[1118]; PBac{y[+mDint2] w[+mC]=UAS-Mpv17.HA}VK00033
99875		y[1] w[1118]; PBac{y[+mDint2] w[+mC]=UAS-dpr7.HA}VK00037
99876		y[1] w[1118]; PBac{y[+mDint2] w[+mC]=UAS-dpr7.HA}VK00033
99877		y[1] w[1118]; PBac{y[+mDint2] w[+mC]=UAS-CG33521.HA}VK00037
99878		y[1] w[1118]; PBac{y[+mDint2] w[+mC]=UAS-CG33521.HA}VK00033
99879		y[1] w[1118]; PBac{y[+mDint2] w[+mC]=UAS-CG33941.HA}VK00037
99880		y[1] w[1118]; PBac{y[+mDint2] w[+mC]=UAS-CG33941.HA}VK00033
Zurich ORFeome Project donated on behalf of FCRP		
95326	118839	y[1] w[*]; M{RFP[3xP3.PB] w[+mC]=UAS-onecut.ORF.3xHA.GW}ZH-86Fb/TM3, Sb[1] Ser[1]
95327	118840	y[1] w[*]; M{RFP[3xP3.PB] w[+mC]=UAS-CG11360.ORF.3xHA.GW}ZH-86Fb
95328	118841	y[1] w[*]; M{RFP[3xP3.PB] w[+mC]=UAS-Nfl.ORF.3xHA.GW}ZH-86Fb/TM3, Sb[1] Ser[1]
95329	118842	y[1] w[*]; M{RFP[3xP3.PB] w[+mC]=UAS-CG32006.ORF.3xHA.GW}ZH-86Fb
95330	118843	y[1] w[*]; M{RFP[3xP3.PB] w[+mC]=UAS-fuss.ORF.3xHA.GW}ZH-86Fb
95331	118844	y[1] w[*]; M{RFP[3xP3.PB] w[+mC]=UAS-anne.ORF}ZH-86Fb
95332	118845	y[1] w[*]; M{RFP[3xP3.PB] w[+mC]=UAS-dati.ORF.3xHA.GW}ZH-86Fb/TM3, Sb[1] Ser[1]
95333	118846	y[1] w[*]; M{RFP[3xP3.PB] w[+mC]=UAS-ey.ORF.3xHA.GW}ZH-86Fb
95334	118847	y[1] w[*]; M{RFP[3xP3.PB] w[+mC]=UAS-pan.ORF.3xHA.GW}ZH-86Fb/TM3, Sb[1] Ser[1]
95335	118848	y[1] w[*]; M{RFP[3xP3.PB] w[+mC]=UAS-pho.ORF.3xHA.GW}ZH-86Fb/TM3, Sb[1] Ser[1
600205		y[1] w[*]; M{RFP[3xP3.PB] w[+mC]=UAS-fd102C.ORF.3xHA.GW}ZH-86Fb/TM3, Sb[1] Ser[1]
600206		y[1] w[*]; M{RFP[3xP3.PB] w[+mC]=UAS-toy.ORF.3xHA.GW}ZH-86Fb
O'Connor lab (Univ. Minnesota) donated on behalf of FCRP		
97108	118853	y[1] w[1118]; P{w[+mC]=UAS-Actbeta.Z}3b2
97109	118854	y[1] w[1118]; P{w[+mC]=UAS-myoglianin.G}4d2
97110	118855	y[1] w[1118]; P{w[+mC]=UAS-myoglianin.G}3
97111	118856	y[1] w[1118]; P{w[+mC]=UAS-mav.G}2
97112	118857	y[1] w[1118]; P{w[+mC]=UAS-mav.G}3
97113	118858	w[1118]; P{w[+mC]=UAS-apolpp.E}2
97114	118859	y[1] w[1118]; P{w[+mC]=UAS-apolpp.E}3/TM2

Table S2B

FCRP: UAS.human cDNA stocks with Bloomington and Kyoto center numbers		
**Bloom #**	**Kyoto #**	**Genotype (60)**
97104	118849	w[1118]; PBac{y[+mDint2] w[+mC]=UAS-hYY1.HA}VK00033
97105	118850	w[1118]; PBac{y[+mDint2] w[+mC]=UAS-hYY1.HA}VK00037
97106	118851	w[1118]; PBac{y[+mDint2] w[+mC]=UAS-hTNRC6A.N}VK00033
97107	118852	w[1118]; Bac{y[+mDint2] w[+mC]=UAS-hNDUFS1.HA}VK00037
94337	118741	y[1] w[1118]; PBac{y[+mDint2] w[+mC]=UAS-hINHBA.N}VK00002
94338	118742	y[1] w[1118]; PBac{y[+mDint2] w[+mC]=UAS-hINHBB.N}VK00002
94339	118743	y[1] w[1118]; PBac{y[+mDint2] w[+mC]=UAS-hINHBC.N}VK00002
94340	118744	y[1] w[1118]; PBac{y[+mDint2] w[+mC]=UAS-hINHBE.N}VK00002
94341	118745	y[1] w[1118]; PBac{y[+mDint2] w[+mC]=UAS-hTGFB1.N}VK00002
94342	118746	y[1] w[1118]; PBac{y[+mDint2] w[+mC]=UAS-hTGFB2.N}VK00002
94343	118747	y[1] w[1118]; PBac{y[+mDint2] w[+mC]=UAS-hTGFB3.N}VK00002
94344	118748	y[1] w[1118]; PBac{y[+mDint2] w[+mC]=UAS-hZFHX2.HA}VK00002
94345	118749	y[1] w[1118]; PBac{y[+mDint2] w[+mC]=UAS-hZFHX3.HA}VK00002
	118978	w[1118]; PBac{y[+mDint2] w[+mC]=UAS-hLIMD2.HA}VK00033
	118979	w[1118]; PBac{y[+mDint2] w[+mC]=UAS-hRNF6.HA}VK00033
	118980	w[1118]; PBac{y[+mDint2] w[+mC]=UAS-hTNRC6C.HA}VK00033
	118981	w[1118]; PBac{y[+mDint2] w[+mC]=UAS-hFBN2.N}VK00033
	118982	w[1118]; PBac{y[+mDint2] w[+mC]=UAS-hSLC39A8.HA.N}VK00033
	118983	w[1118]; PBac{y[+mDint2] w[+mC]=UAS-hTAF3.HA}VK00033
	118984	w[1118]; PBac{y[+mDint2] w[+mC]=UAS-hMEX3C.HA}VK00033
	118985	w[1118]; PBac{y[+mDint2] w[+mC]=UAS-hFBLN2.HA}VK00033
	118986	w[1118]; PBac{y[+mDint2] w[+mC]=UAS-hNDUFS1.HA}VK00033
	118987	w[1118]; PBac{y[+mDint2] w[+mC]=UAS-hTGFB3.N}VK00033
	118988	w[1118]; PBac{y[+mDint2] w[+mC]=UAS-hTGFB2.N}VK00033
	118989	w[1118]; PBac{y[+mDint2] w[+mC]=UAS-hTGFB1.N}VK00033
	118990	w[1118]; PBac{y[+mDint2] w[+mC]=UAS-hINHBA.N}VK00033
	118991	w[1118]; PBac{y[+mDint2] w[+mC]=UAS-hINHBB.N}VK00033
	118992	w[1118]; PBac{y[+mDint2] w[+mC]=UAS-hINHBC.N}VK00033
	118993	w[1118]; PBac{y[+mDint2] w[+mC]=UAS-hINHBE.N}VK00033
	118994	w[1118]; PBac{y[+mDint2] w[+mC]=UAS-hZFHX2.HA}VK00033
	118995	w[1118]; PBac{y[+mDint2] w[+mC]=UAS-hZFHX3.HA}VK00033
	118996	w[1118]; PBac{y[+mDint2] w[+mC]=UAS-hONECUT1.HA}VK00033
	118997	w[1118]; PBac{y[+mDint2] w[+mC]=UAS-hTNRC6A.HA}VK00037
98424	118998	w[1118]; PBac{y[+mDint2] w[+mC]=UAS-hLIMD2.HA}VK00037
98425	118999	w[1118]; PBac{y[+mDint2] w[+mC]=UAS-hRNF6.HA}VK00037
98426	119000	w[1118]; PBac{y[+mDint2] w[+mC]=UAS-hTNRC6C.HA}VK00037
98427	119001	w[1118]; PBac{y[+mDint2] w[+mC]=UAS-hFBN2.N}VK00037
98428	119002	w[1118]; PBac{y[+mDint2] w[+mC]=UAS-hSLC39A8.HA.N}VK00037
98429	119003	w[1118]; PBac{y[+mDint2] w[+mC]=UAS-hTAF3.N}VK00037
98430	119004	w[1118]; PBac{y[+mDint2] w[+mC]=UAS-hMEX3C.HA}VK00037
98431	119005	w[1118]; PBac{y[+mDint2] w[+mC]=UAS-hFBLN2.HA}VK00037

98434	119006	w[1118]; PBac{y[+mDint2] w[+mC]=UAS-hONECUT1.HA}VK00037
	119007	w[1118]; PBac{y[+mDint2] w[+mC]=UAS-hLIMA1.N}VK00033
	119008	w[1118]; PBac{y[+mDint2] w[+mC]=UAS-hVPS72.HA}VK00033
	119009	w[1118]; PBac{y[+mDint2] w[+mC]=UAS-hCADPS2.N}VK00033
	119010	w[1118]; PBac{y[+mDint2] w[+mC]=UAS-hSYT5.HA}VK00033
	119011	w[1118]; PBac{y[+mDint2] w[+mC]=UAS-hINHA.N}VK00033
	119012	w[1118]; PBac{y[+mDint2] w[+mC]=UAS-hARHGAP36.HA}VK00033
	119013	w[1118]; PBac{y[+mDint2] w[+mC]=UAS-hGLI2.N}VK00033
	119014	w[1118]; PBac{y[+mDint2] w[+mC]=UAS-hTRIP6.HA}VK00033
	119015	w[1118]; PBac{y[+mDint2] w[+mC]=UAS-hZYX.HA}VK00033
600291	119016	w[1118]; PBac{y[+mDint2] w[+mC]=UAS-hLIMA1.N}VK00037
600292	119017	w[1118]; PBac{y[+mDint2] w[+mC]=UAS-hVPS72.HA}VK00037
	119018	w[1118]; PBac{y[+mDint2] w[+mC]=UAS-hCADPS2.N}VK00037
600294	119019	w[1118]; PBac{y[+mDint2] w[+mC]=UAS-hSYT5.HA}VK00037
600295	119020	w[1118]; PBac{y[+mDint2] w[+mC]=UAS-hINHA.N}VK00037
	119021	w[1118]; PBac{y[+mDint2] w[+mC]=UAS-hARHGAP36.HA}VK00037
600293	119022	w[1118]; PBac{y[+mDint2] w[+mC]=UAS-hGLI2.N}VK00037
	119023	w[1118]; PBac{y[+mDint2] w[+mC]=UAS-hTRIP6.HA}VK00037
600296	119024	w[1118]; PBac{y[+mDint2] w[+mC]=UAS-hZYX.HA}VK00037

Table S3

FCRP: 4th recombination and FRT101F mutagenized stocks with Bloomington and Kyoto stock numbers		
**Bloom #**	**Kyoto #**	**Genotype (12)**
**Meiotic recombination**		
94593	118750	y[1] w[67c23]; Blm[N1] rec[2] P{UASp-Blm.K}3/TM6C, Sb[1]; TI{GMR-HMS04515}Gat[eya]/In(4)ci[D], ci[D] pan[ciD]
94594	118751	y[1] w[67c23]; Blm[D2] ry[*] rec[1] Ubx[bx-34e] P{w[+mC]=matalpha4-GAL-VP16}V37/TM6C, Sb[1]; TI{GMR-HMS04515}Gat[eya]/In(4)ci[D], ci[D] pan[ciD]
**Mitotic recombination**		
94595	118752	w[1118]; TI{RFP[DsRed.3xP3.cUa]=TI}FRT101F-DsRed+
94596	118753	w[1118]; TI{TI}FRT101F
94597	118754	w[1118]; TI{RFP[DsRed.3xP3.cUa]=FRT.Tub.GAL80.O}101F-DsRed+
94598	118755	w[1118]; TI{FRT.Tub.GAL80.O}101F
**FRT101 mutagenized**		
600261		y[1] w[1118]; P{w[+mC]=Act5C-GAL4}25FO1, P{w[+mC]=UAS-GFP.U}2/SM6a; TI{TI}FRT101F zfh2[51A]/TI{GMR-HMS04515}Gat[eya]
600262		y[1] w[1118]; TI{TI}FRT101F apolpp[B]/In(4)ci[D], ci[D] pan[ciD]
600263		y[1] w[1118]; TI{TI}FRT101F Pur-alpha[B]/In(4)ci[D], ci[D] pan[ciD]
		y[1] w[1118]; TI{TI}FRT101F Ephrin[A]
		y[1] w[1118]; TI{TI}FRT101F ND-49[A]/In(4)ci[D], ci[D] pan[ciD]
		y[1] w[1118]; TI{TI}FRT101F Slip1[A]

Table S4

FCRP & GDP: Phenotypes for stocks from 4th coding genes in 5 sets (FCRP data in red & GDP in black except UAS.fly cDNA)						
**Genes proximal to distal long arm**	**Flybase gene ID**	**UAS.fly cDNAs inserted chromosome**	**T2A.GAL4 gene traps homo lethal/viable**	**eGFP protein traps homo lethal/viable**	**UAS.human cDNAs human gene symbols**	**New mutations on FRT101F homo lethal/viable**
Jyalpha	FBgn0267363	pipeline	lethal	lethal		pipeline
PlexB	FBgn0052009	II/III	lethal	viable		pipeline
ci	FBgn0265633	II/III	lethal	viable	GLI2	pipeline
RpS3A	FBgn0264617	published	pipeline	pipeline		pipeline
pan	FBgn0263851	II/III	lethal	viable	LEF1, TCF7L1	lethal
Ank	FBgn0085432	published	viable	viable	ANK1, ANK3	viable
anne	FBgn0011747	III	lethal	viable	ATP13A3	lethal
CG32006	FBgn0264616	III	lethal	pipeline		pipeline
CG31997	FBgn0052006	II/III	viable	viable	not conserved	pipeline
CG33978	FBgn0051997	pipeline	viable	lethal	not conserved	lethal
Arl4	FBgn0266725	II/III	viable	viable		viable
CG2316 (Abcd1)	FBgn0039889	II/III	viable	viable		viable
CG31998	FBgn0265634	pipeline	viable	viable		lethal
Crk	FBgn0051998	II	lethal	pipeline	CRK, CRKL	pipeline
CG31999	FBgn0024811	II/III	viable	viable	FBN2, FBLN2	viable
yellow-h	FBgn0051999	II/III	viable	viable		viable
CG1674	FBgn0267734	II/III	viable	viable		pipeline
dpr7	FBgn0039897	II/III	lethal	viable		viable
RhoGAP102A	FBgn0053481	pipeline	viable	viable	ARHGAP6, ARHGAP36	viable
NfI	FBgn0259216	III	lethal	viable	NFIB	pipeline
Syt7	FBgn0264793	published	viable	viable	SYT1, SYT2, SYT5	viable
Rad23	FBgn0039900	published	viable	viable	RAD23A, RAD23B	viable
Zip102B	FBgn0026777	published	viable	pipeline	SLC39A8, SLC39A9	pipeline
CG32850 (Rnf11)	FBgn0039902	II/III	lethal	lethal	RNF6, RNF11	pipeline
PMCA	FBgn0052850	pipeline	lethal	viable	ATP2B2, ATP2B4	lethal
Hcf	FBgn0259214	III	lethal	viable	HCFC2, KLHDC4	lethal
dati	FBgn0039904	III	lethal	lethal		pipeline
Igs	FBgn0264794	II/III	lethal	viable		viable

CaMKI	FBgn0039907	II/III	viable	viable	CAMK1D, CAMK1G, CAMK2A, 2B, 2D, PNCK	pipeline
bip2 (Taf3)	FBgn0266619	II/III	pipeline	pipeline	TAF3, TAF8	pipeline
CG33941	FBgn0026262	II/III	viable	viable	not conserved	TRiP making transgene
Asator DH.0		not applicable	viable	viable	not applicable	not applicable
Asator DH.2	FBgn0262731	published	lethal	viable	TTBK1, TTBK2	pipeline
zfh2	FBgn0039908	pipeline	lethal	lethal	ZFHX2, ZFHX3	lethal
Thd1	FBgn0004607	pipeline	lethal	viable		viable
Pur-alpha	FBgn0264618	II	viable	viable	PURG	viable
ND-49	FBgn0022361	II/III	lethal	lethal		viable
Ephrin	FBgn0039909	pipeline	lethal	pipeline	NDUFS1	viable
CG1909	FBgn0040324	II/III	viable	viable	RAPSN	pipeline
onecut	FBgn0266726	III	lethal	lethal	ONECUT1	pipeline
Eph	FBgn0028996	III	viable	viable	EPHA1, EPHA2, EPHA8, EPHB1, EPHB2, EPHB6	pipeline
mav	FBgn0025936	II/III	viable	viable	TGFB1, TGFB2, TGFB3	viable
Gat	FBgn0262302	II/III	viable	viable	SLC6A5, SLC6A15, SLC6A19, SLC6A20	pipeline
Ekar	FBgn0039915	II / III	viable	viable	GIRK5	viable
gw	FBgn0264820	published	lethal	lethal	TNRC6A,TNRC6C	pipeline
Slip1	FBgn0051992	pipeline	lethal	viable		viable
CG11360	FBgn0024728	III	viable	viable	MEX3C	viable
myo	FBgn0039920	II/III	lethal	lethal	GDF11, MSTN	lethal
ey	FBgn0026199	II/III	lethal	viable	PAX6, PAX7, PITX3, RAX, LHX6, GSC	lethal
bt	FBgn0005558	pipeline	viable	viable		pipeline
MED26	FBgn0266727	pipeline	lethal	viable		viable
Sox102F	FBgn0039923	III/X	lethal	pipeline		viable
fd102C	FBgn0039938	published	pipeline	pipeline	FOXS1	pipeline
Gyf	FBgn0266616	III/X	lethal	lethal		pipeline
unc-13	FBgn0263344	published	viable	viable		pipeline
eIF4G1	FBgn0263093	pipeline	lethal	lethal		pipeline
mGluR DH.1		not applicable	viable	viable	not applicable	not applicable
mGluR DH.2	FBgn0023213	published	viable	viable		pipeline
4E-T	FBgn0019985	II/III	viable	viable		lethal

fuss MI13731		not applicable	viable	not applicable	not applicable	not applicable
fuss MI03207	FBgn0264822	III	viable	viable		pipeline
toy	FBgn0083990	II/III	lethal	viable	Same genes ey above	lethal
PlexA	FBgn0019650	III	lethal	lethal	PLXNA3	pipeline
CG11077	FBgn0264823	II/III	pipeline	pipeline	MPV17	pipeline
CG11076	FBgn0039930	II/III	pipeline	pipeline	not conserved	pipeline
ATPsynbeta	FBgn0039929	published	lethal	lethal	ATP5F1	pipeline
CaMKII	FBgn0010217	II	lethal	viable	Same genes CaMKI above	pipeline
Zyx	FBgn0264607	II	lethal	viable	LPP, ZYX, TRIP6	pipeline
apolpp	FBgn0011642	II/III	lethal	lethal	APOE2, APOE3, APOE4	lethal
Actbeta	FBgn0087002	II/III	lethal	viable	INHA, INHBA, INHBB, INHBC, INHBE	pipeline
sv	FBgn0024913	II/III	lethal	lethal	Same genes ey above	lethal
Cals	FBgn0005561	II/III	viable	pipeline		pipeline
Arf102F	FBgn0039928	II/III	pipeline	pipeline	ARF5	lethal
CG11155 DH.1		not applicable	lethal	pipeline	not applicable	not applicable
CG11155 DH.0	FBgn0013749	II/III	lethal	viable	GRIK5	viable
CG32017	FBgn0039927	II/III	viable	viable	not conserved	pipeline
Kif3C	FBgn0052017	II/III	viable	viable		lethal
pho	FBgn0039925	II/III	lethal	viable	YY1	pipeline
CG33521	FBgn0002521	II/III	viable	viable	LIMD2. LIMA1	viable
PIP4K	FBgn0250819	published	lethal	lethal	PIP4K2A, PIP5K1A	TRiP making transgene
Mitf	FBgn0039924	published	lethal	pipeline	TFEB, TFE3, MITF, SREBF2	lethal
Dyrk3	FBgn0263112	II/III	viable	pipeline	DYRK2	pipeline
Cadps	FBgn0027101	pipeline	lethal	viable	CAPDS2	pipeline
